# Day-to-day Social Interactions Online and Offline: The Interplay Between Interaction Mode, Interaction Quality, and Momentary Well-being

**DOI:** 10.1177/00936502251341088

**Published:** 2025-05-31

**Authors:** Timon Elmer, Aurelio Fernández, Jeffrey A. Hall, Marie Stadel

**Affiliations:** 1 Department of Psychology, Applied Social and Health Psychology, University of Zurich, Zurich, Switzerland; 2 Ghent University, imec-mict, Ghent, Belgium; 3 School of Communication, University of Navarra, Pamplona, Spain; 4 Institute for Culture and Society, University of Navarra, Pamplona, Spain; 5 University of Kansas, Lawrence, KS, USA; 6 Department of Sociology, University of Groningen, The Netherlands; 7 Department of Psychometrics and Statistics, University of Groningen, The Netherlands

**Keywords:** social interaction, computer-mediated communication, interaction quality, well-being, ecological momentary assessment

## Abstract

Digital social interactions differ in many ways from face-to-face interactions. This study examines four preregistered hypotheses on the within-person interplay between interaction mode (i.e., digital vs. face-to-face interactions), interaction quality, and momentary well-being. Young adults from Spain (*N*_1_ = 216) and the Netherlands (*N*_2_ = 22)—provided 5,116 and 1,386 Ecological Momentary Assessments (EMA), respectively. In the Spanish sample, there were no differences in interaction quality between digital and face-to-face interactions, whereas in the Dutch sample, digital interactions were of higher quality. Interaction quality was positively associated with momentary well-being in both samples. Momentary well-being was higher after face-to-face interactions in the Spanish but not in the Dutch sample. Interaction quality did not mediate the relationship between interaction mode and well-being; instead, it moderated it in the Spanish sample. Although interaction quality was consistently associated with momentary well-being, it only partially explains why face-to-face interactions differ from digital ones.

## Introduction

Social interactions are a fundamental aspect of human life, serving as a means of communication, connection, and support. Research has consistently demonstrated the importance of social interactions for physical and mental health outcomes (e.g., Elmer, Ram, et al., 2023; Holt-Lunstad et al., 2010, 2015). However, technological developments have led to a major shift in the way we interact with one another, with an increasing number of social interactions taking place through digital media (Hall, 2020b). A significant percentage of adults in Western societies now regularly engage in daily digital interactions, such as phone calls, video calls, or text messaging (Hall, Dominguez, et al., 2023). The choice of the interaction mode—whether people interact digitally or Face-to-Face (FtF)—appears to be related to how they feel after these interactions (Goodman-Deane et al., 2016; Hall, 2020a; Lin & Lachman, 2021; Macdonald et al., 2021). Yet the mechanisms by which the mode of interaction affects momentary well-being is not fully understood.

One possible pathway is through interaction quality. Interaction quality is defined as an individual’s subjective evaluation of the positive characteristics related to a specific social interaction (Fernández, Vanden Abeele, Sádaba et al., 2025). Due to the reduction of physical cues, communication theories such as social presence theory (Short et al., 1976) and media richness theory (Daft & Lengel, 1986), initially suggested that digitally-mediated social interactions may exhibit lower interaction quality, which, in turn, may be associated with how individuals feel after these interactions (i.e., exhibiting different levels of momentary well-being; Hülür et al., 2023). However, more recent theoretical accounts (e.g., Walther & Parks, 2002) have challenged the primacy of face-to-face communication, as we discuss further below. The goal of the present investigation is to examine the role of interaction quality as a mediator between the relationship between interaction mode (i.e., FtF vs. digital) and momentary well-being. Understanding how social interaction quality is associated with individuals’ momentary well-being can help to explain what kind of daily social practices—both FtF and digital—contribute to long-term well-being and life satisfaction (Hall & Merolla, 2020; Wichers, 2014).

In the remainder of this introduction, we discuss social interactions and their psychosocial effects on daily life. Then, we discuss the theoretical and empirical relationships between (a) the mode of interaction (i.e., FtF vs. digital) and interaction quality, (b) interaction quality and momentary well-being, and (c) mode of interaction and momentary well-being. From these arguments, four hypotheses were derived and preregistered prior to data analysis (OSF, https://osf.io/dezuw/).

### Studying Online and Offline Social Interaction in Daily Life

Ecological momentary assessments (EMAs) are an increasingly popular method to study how people experience social interaction in their daily lives (Trull & Ebner-Priemer, 2014). EMAs require participants to complete repeated surveys on their smartphones as they go about their daily lives. Compared to global, retrospective self-reports of well-being and social interaction characteristics (Brissette et al., 2000; Cooke et al., 2016), EMAs minimize retrospective biases, providing a more accurate depiction of the nuanced fluctuations in feelings and experiences during and after daily social interactions (Reis & Wheeler, 1991; Trull & Ebner-Priemer, 2014). EMA is particularly suitable for assessing the qualitative and subjective features of social experiences that vary from interaction to interaction (Langener et al., 2023; Reis & Wheeler, 1991). Historically, EMA research has focused on the effects of offline FtF interactions on momentary well-being, whereas digital social interactions have received less attention (Liu et al., 2019).

Additionally, EMA data can be used to differentiate the within- and between-person effects. By examining how one momentary assessment differs from how the same person usually behaves, researchers can study the within-person interplay between variables (Bolger & Laurenceau, 2013). EMA studies have examined interaction mode, interaction quality, and well-being, but have typically neglected the importance of differentiating within- from between-person effects—with some recent exceptions (Achterhof et al., 2022; Hülür et al., 2023; Macdonald et al., 2021). In this study, we focus on examining the within-person effects, as these can test the micro-mechanisms by which interaction mode, quality, and momentary well-being relate to one another moment-by-moment.

### Relationship Between Mode of Interaction and Interaction Quality

For decades, social interaction quality has been a key focus in research on computer-mediated communication. Early dominant theories, known collectively as cues-filtered-out theories (Walther & Parks, 2002), posited that digitally mediated communication lacks non-verbal cues, which are “filtered out,” limiting the modality’s ability to perform the social functions conveyed by those cues. As a result, these theories argued that FtF interactions, which offer richer communication through both verbal and non-verbal cues, are inherently of higher quality than digitally mediated interactions. For example, social presence theory (Short et al., 1976) suggests that different communication modes vary in their capacity to transmit nonverbal cues, which in turn affects interaction quality. According to this theory, digital communication modes that convey more cues are thought to approximate the quality of FtF interactions. Similarly, media richness theory (Daft & Lengel, 1986) proposes that the effectiveness of a communication mode depends on its ability to transmit nuanced and ambiguous information. Because digital communication struggles to convey certain nonverbal cues (e.g., emotional expressions), it is associated with lower interaction quality.

However, over the past two decades, cues-filtered-out theories have faced increasing criticism (Ishii et al., 2019; Walther, 2011). Initially developed for organizational contexts, empirical research in interpersonal settings proposed that individuals can adapt to the limitations of mediated communication and may even prefer fewer cues in certain situations (Walther, 2011), resonating with early findings showing that computer-mediated communication can support socioemotional content (Rice & Love, 1987). This shift gave rise to cues-filtered-in theories (Walther & Parks, 2002), such as social information processing theory (Walther, 1992) and the hyperpersonal model (Walther, 1996). These theories emphasize that digitally mediated interactions can reach, or even surpass, the quality of FtF interactions, as individuals may learn to adapt to the cues afforded by digitally mediated modes (Walther, 2011). In some cases, the absence of nonverbal cues may even enhance communication, as the hyperpersonal model suggests (Walther, 1996). For example, self-disclosure could increase when an interaction partner is anonymous and physically distant (Trepte et al., 2018).

In sum, there is no theoretical consensus as to whether digital social interactions should be of lower or higher quality than FtF interactions (Hall, 2020b). Empirical research on daily life social interactions, however, has shown the primacy of FtF interactions in quality over digital interactions (Hall, Pennington, Merolla, 2023). At the same time, prior empirical research consistently demonstrates that digital interactions differ from FtF interactions in various quality-related aspects showing, for example, that self-disclosure was higher in FtF than in digitally mediated interactions (Ruppel et al., 2017), that engaging in FtF interactions was associated with more positive impressions of their partner than in digitally mediated interactions (Okdie et al., 2011), and that text-based interactions were lower in valence and social relatedness compared to FtF interactions, whereas phone calls were rated higher in meaningfulness than FtF interactions (e.g., Hülür et al., 2023). Hence, more research on how and why quality varies between FtF and digital social interactions, including different empirical contexts (e.g., among young adults) and contextual variables (e.g., interaction partner closeness), can help inform further theory development.

Particularly, among young adults, who are both prevalent and comfortable users of mediated communication, FtF is still considered as more useful for expressing a wide range of emotions and accomplishing various goals; it has been associated with higher levels of enjoyment and supportiveness in daily interactions (Bayer et al., 2021), and it is *perceived* as the highest quality form of communication by 83% of young adults (Duran & Kelly, 2017; Eden & Veksler, 2016). Although FtF and digital interactions have been compared in their effects on different outcomes, including momentary affect (e.g., Achterhof et al., 2022; Macdonald et al., 2021), there has been less effort to link these differences to social interaction quality specifically.

In this study, we contribute to research and theory development by examining the within-person interplay between interaction mode and interaction quality. In doing so, we operationalize social interaction quality using two approaches. First, we adopt a theory-driven, deductive definition of quality which highlights the importance of valuing and feeling valued during the interaction (Fernández, Vanden Abeele, Sádaba et al., 2025). Second, we take an inductive bottom-up approach, using a broader range of quality features that resulted from a focus group discussion (e.g., enjoyment, meaningfulness; see Methods section). Using these two operationalizations, we examine whether there are systematic differences in the quality of social interactions between different modes of interaction on a within-person level among young adults. We expect digital interactions to be of lower quality because this aligns with previous findings (Hall, Pennington, Merolla, 2023). Thus, our first hypothesis (H1) is:

Digital interactions are associated with a lower level of perceived interaction quality than face-to-face interactions on a within-person level.

### Relationship Between Social Interaction Quality and Momentary Well-Being

Numerous theories make the implicit or explicit assumption that higher quality interactions result in higher well-being. For example, social support theory (Cohen, 2004; Cohen & Ashby, 1985; Lakey & Orehek, 2011) argues that well-being is maintained and sustained when individuals perceive higher social support during interactions. Similarly, self determination theory (Deci & Ryan, 2012; Ryan & Deci, 2000) posits that higher well-being is linked to feelings of connection and relatedness, which are fostered through engaging in high-quality interactions (Fernández, Elmer, Sádaba, et al., 2025; Sun et al., 2019). Communicate bond belong theory (Hall, 2018a; Hall & Davis, 2017) suggests that high-quality communication content influences individuals’ ability to get their need to belong met, which increases well-being.

In line with these theoretical accounts, momentary well-being (e.g., measured as affect) is higher after interactions with lower levels of conflict (Finch et al., 1999; Ilies et al., 2011; Rook, 2001) and higher levels of enjoyment, depth, pleasantness, self-disclosure, and importance (Bernstein et al., 2018; Berry & Hansen, 1996; Elmer, Ram, Gloster, et al., 2023; Sun et al., 2019; Vogel et al., 2017). Yet, there has been little research examining the relationship between interaction quality and momentary well-being in the context of digital interactions in daily life settings and on a within-person level (Liu et al., 2019). In this study, we propose Hypothesis 2 (H2): *Interaction quality is positively associated with subsequent well-being (i.e., immediately after the social interaction) on a within-person level.*

### Relationship Between Mode of Interaction and Momentary Well-Being

Although decades of research has emphasized the importance of FtF interactions for well-being (Diener, 1984; Elmer, Geschwind, Peeters, et al., 2020; Kahneman et al., 2004; Macdonald et al., 2021), it is less clear whether digital interactions are similarly associated with momentary well-being. In line with *cues-filtered-out* and *cues-filtered-in* theories, the association between digital interactions and momentary well-being may derive from the specific perceived affordances provided by different digital interactions (Fox & McEwan, 2017). Recent between-person investigations suggest that each modality has specific benefits, but that they differ in their association with life satisfaction and loneliness. For instance, frequent FtF interactions are associated with higher life satisfaction and lower loneliness, while video chats and voice calls also contribute positively to life satisfaction, with voice calls reducing loneliness through greater relationship maintenance satisfaction (Hall, Dominguez, Mihailova, 2023). Moreover, although digital communication can alleviate social disconnection, it may not fully satisfy belongingness needs, as some modalities (e.g., texting, social media) are less effective in fostering emotional bonds compared to FtF interactions (Hall, Pennington, Merolla, 2023). In examining within-person effects specifically, a higher ratio of FtF interactions to digital interactions during a specific day is associated with higher end-of-the-day well-being (Lin & Lachman, 2021; Macdonald et al., 2021). Compared to digital interactions, individuals report higher affect valence, higher positive affect, and lower negative affect and loneliness after FtF interactions (Achterhof et al., 2022; Kafetsios et al., 2017; Kroencke et al., 2023; Kushlev & Heintzelman, 2018; Roshanaei et al., 2023). Given this consistency in the literature about interaction mode and momentary well-being, we hypothesize that (H3): *Digital interactions are associated with a lower level of subsequent momentary well-being (i.e., lower levels of positive affect indicators and higher levels of negative affect indicators) than face-to-face interactions on a within-person level.*

### Interaction Quality as a Mediator Between Interaction Mode and Well-Being

Empirical evidence supports two key claims: First, that high-quality social interactions are positively associated with well-being, and second, that face-to-face (FtF) interactions, compared to digital communication, are more strongly associated with higher well-being (e.g., Hall, Pennington, et al., 2023; Sun et al., 2019). In the present investigation we focus on the potential mediating effect of interaction quality in explaining the association between interaction mode and well-being. Through this indirect effect, FtF interactions may surpass digital interactions in quality (H1), which potentially leads to heightened well-being after FtF interactions (H2). Consistent with prior research, we anticipate differences in well-being measures across interaction modes (H3), which may diminish when accounting for the influence of interaction quality on well-being. Therefore, our fourth hypothesis (H4) explores the mediating effect of interaction quality: *The within-person association between interaction mode and momentary well-being is mediated by interaction quality.*

### The Present Study

**Figure 1.**
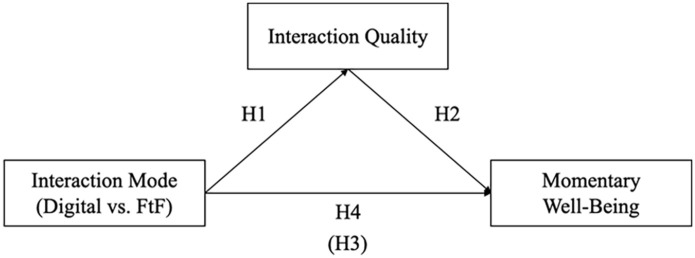
Illustration of the three key variables and their hypothesized relations. *Note.* H1-H4; Hypotheses 1-4, Hypothesis in bracket refers to the total effect (i.e., absence of mediator). FtF; Face-to-Face interaction. Digital interactions entail all interaction modes other than FtF.

Figure 1 shows an illustration of the four preregistered hypotheses^1^. This study tests this theoretical model in two samples of young adults from Spain (*N*_1_ = 216; *T*_1_ = 5,116) and the Netherlands (*N*_2_ = 22; *T*_2_ = 1,386), with differing levels of granularity in social interaction modes, quality, and well-being measures. The two samples differ in four key ways: First, the Spanish sample used a signal-contingent EMA design, whereas the Dutch sample additionally incorporated event-contingent assessments, capturing a broader range of interactions (Stadel, Van Duijn, Wright, et al., 2024). Second, the Spanish sample assessed social interaction quality based on feelings of being valued, whereas the Dutch sample measured dimensions such as enjoyment and meaningfulness. Third, momentary well-being was measured using single-item assessments of affect valence and loneliness in the Spanish sample, whereas the Dutch sample used multi-item scales for positive and negative affect. Finally, the Dutch sample includes measures of interaction partner closeness and the number of unique interaction partners, factors that can explain up to 20% of the variance in interaction quality (Stadel, Van Duijn, Wright, et al., 2024) and serve as important control variables. The use of these different designs contributes to the methodological rigor of our study, allowing for a conceptual replication of findings while testing our hypotheses and accounting for key relational and contextual variables.

## Methods

### Participants

#### Spanish Sample

The Spanish sample initially consisted of *N* = 256 participants with 5,635 interaction reports. We removed seven participants with a total of 17 reported interactions because they did not comply with the requirement of reporting five or more interactions registered per day. We additionally removed 33 individuals from the dataset (500 interactions) because they did not show variance in their reports on at least one of the key variables (i.e., interaction mode, interaction quality items, affect valence, loneliness) (e.g., they always answered the lowest possible score in the affect valence item). The final set consisted of *N_S_* = 216 individuals with 5,116 observations. The sample was predominantly female (*N* = 182, 84%) with a mean age of 23.12 (*SD* = 1.87). One percent of the participants (*n* = 3) categorized themselves as lower socioeconomic class, 29% (*n* = 63) in lower-middle, 58% (*n* = 126) in middle, and 11% (*n* = 24) in upper-middle. Forty-six percent of the participants (*n* = 99) were single and 54% (*n* = 117) were in a romantic relationship. Of the sample, 17% (*n* = 36) were living as a couple and the other 83% (*n* = 180) were living either with their family, sharing a flat or in a residence, or alone. 38% of the participants (*n* = 82) were only studying, 24% (*n* = 51) were studying and working, and the remaining 42% (*n* = 83) were working, looking for a job, or in other situations.

#### Dutch Sample

In the Dutch sample, there were *N* = 23 individuals with *T* = 1,685 interaction reports. We removed 239 observations because the reported interactions did not take place within the last hour of the EMA survey. We additionally removed another observation because of a missing negative affect score. We additionally removed all observations from one participant from the dataset because the person did not show variation in the mode variable (i.e., only face-to-face interactions were reported). The final sample consisted of *N_D_* = 22 participants with *T_D_* = 1,386 social interaction observations. The majority of the sample identified as female (*N* = 17, 77%) and did not live in the same country as their family (*N* = 17, 77%) (i.e. they were international students). The mean age was 21.41 (*SD* = 2.87).

### Procedure

#### Spanish Sample

Data for the Spanish sample was collected between March 21st and 30th 2022 in Spain by an external company (Netquest). Inclusion criteria for the study were (a) being between age 18 and 25, (b) owning an Android smartphone, (c) being resident in Spain. Participants were provided with a link to a webpage where they could learn more about the study, give the informed consent, and follow the instructions for installing the Ethica App, which was used to collect EMA responses. To incentivize participation, participants received a voucher, proportional to their participation rate with a maximum amount equivalent to €55, to obtain products offered by the external company at the end of the study.

Four types of surveys were administered during the course of this study: A baseline survey, weekly surveys, daily surveys, and signal-contingent EMA surveys. Given that our study only uses data from the EMA surveys, we refrain from describing the content of other survey types. More details on the procedure and study design can be found on the OSF page of the data collection and in existing publications stemming from this data collection (Fernández, Elmer, Sádaba, et al., 2025; Fernández, Vanden Abeele, Sádaba, et al., 2025). The ethics committee of the University of Navarra reviewed and approved this procedure (project number: 2021.191).

#### Dutch Sample

Data for the Dutch sample was collected between October 25th and December 2nd 2021 in the Netherlands. During that time, a number of COVID-19-related measures were in place.^2^ Participants were recruited via a university-wide platform that facilitates study participation. The platform is predominantly—but not solely—utilized by students. Following recruitment, participants were asked to complete an online baseline questionnaire including demographic variables, general well-being, and social factors (e.g., how far away they live from their core family). More details about the specific items and the data collection can be found on the OSF page of the project and in existing publications stemming from this data collection (Langener, Bringmann, Kas, et al., 2024; Langener, Stulp, Jacobson, et al., 2024; Stadel, Bringmann, Stulp, et al., 2024; Stadel et al., 2023).

After filling out the baseline questionnaire, participants attended an approximately one-hour instruction session, comprehensively explaining the study’s procedures and its objectives, and instructions for using the Ecological Momentary Assessment (EMA) surveys. Informed consent was acquired from all participants during this instruction session. The core of the data collection consisted of a 28-day EMA period. In the EMA period, participants were instructed to log every face-to-face, video, or phone call interaction they had that lasted longer than 5 min in an event-contingent manner. For a conceptual definition of social interactions, we used Hall’s (2018b) definition of a focused social interaction.^3^ In addition to these event-contingent assessments of social interactions, participants also received six daily signal-contingent EMA surveys (one in the morning, one in the evening, and four at semi-random time points during the day). In these surveys, we further asked whether the participant forgot to indicate any social interactions since the last EMA survey and provided the opportunity to back-log these interactions. Participants were compensated with 60€ for their participation. The ethics committee of the University of Groningen reviewed and approved this study (research codes: PSY-2021-S-0486).

### Materials

A tabular overview over the used measures in the different samples is provided in the Supplemental Table S11.

### Spanish sample

#### Interaction Mode

Interaction mode was assessed with the following item: “How have you conducted the most recent social interaction that happened within the last 10 minutes?”, with the categories “none,” “phone call,” “video call,” “face-to-face,” “social media,” or “text or chat.”

#### Interaction Quality

Interaction quality was measured with the following three items: “Did you feel valued by your interaction partner in your most recent social interaction?,” “Do you think your interaction partner felt valued by you in your most recent social interaction?,” and “Do you consider that after your most recent social interaction, the relationship with your interaction partner. . .”. The first two items were rated on a 7-point scale from “not at all” (1) to “very much” (7), whilst the scale of the third was from “worsened a lot” (1) to “strengthened a lot” (7). This theory-driven approach of operationalizing social interaction quality is based Buber’s (1970) concept of the mutual valuation of oneself and the other, as well as the resulting influence on the relationship itself. It emphasizes the mutual contributions of the individuals involved in the interaction (Fernández, Vanden Abeele, Sádaba, et al., 2025). These three items entered the analyses as manifest variables of a latent interaction quality measure. For robustness and exploratory analyses, we also used a manifest mean score of the three items. Reliability of the mean score was estimated to be ω_within_ = .84 on a within-person level and ω_between_ = .95 on a between-person level.

*Momentary well-being*: Two items were used to measure momentary well-being. A single item measured affect: The item “How do you feel right now?” was rated on a scale from 1 to 7 from “very bad” (1) to “very good” (7). Single-item affect ratings generally provide good agreement with multiple-item scores of the same construct (Verster et al., 2021). Loneliness was measured with the single item: “How lonely do you feel at the moment?”, rated on a scale from 1 to 7 from “not at all” (1) to “very much” (7).

#### Control Variable

In our analyses, we additionally control for gender, which was measured in the baseline survey.

### Dutch Sample

#### Interaction Mode

Interaction mode was measured with an item asking “Which type of interaction did you have?” and the following answer options: “face-to-face,” “video call,” and “phone call.”

#### Interaction Quality

The following items on perceptions of social interaction qualities were measured on an 11-point Likert scale (0 = “strongly disagree” to 10 = “strongly agree”). Self-enjoyment: “I enjoyed the interaction”; Other-enjoyment: “I felt like my interaction partner(s) enjoyed the interaction.”; Meaningfulness: “This interaction was meaningful for me.”; Authenticity: “During this interaction I could be myself.” This set of items was developed based on a focus group discussion during which four psychotherapy patients were asked to reflect on what constitutes a good social interaction and how this can be measured with EMA items. The resulting items reflect the most important dimensions to these four patients. These inductively-derived items have parallels with commonly-used measures of characteristics of interactions. Enjoyment items have been frequently used in the widely-known Rochester Interaction Record (e.g., Reis & Wheeler, 1991). The meaningfulness item is similar to the conversational depth item used by Sun et al. (2019) and the same item was used recently by Hülur et al. (2023). The authenticity item is similar to the item used by Silva et al. (2023) to describe interactions at the end of the day. The reliability of the four items together was ω_within_ = .82 on a within-person level and ω_between_ = .89 on a between-person level.

#### Momentary Well-being

Positive affect was measured with three items (“I feel happy,” “I feel energetic,” and “I feel relaxed”) on an 11-point Likert scale (0 = “*strongly disagree*” to 10 = “*strongly agree*”). The reliability of this scale was ω_within_ = .68 on a within-person level and ω_between_ = .79 on a between-person level. Negative affect was assessed with four items (“I feel sad,” “I feel anxious,” “I feel stressed,” and “I feel irritated.”), rated on an 11-point Likert scale (0 = “strongly disagree” to 10 = “strongly agree”). Reliability of the mean score was estimated to be ω_within_ = .72 on a within-person level and ω_between_ = .92 on a between-person level.

#### Number of Interaction Partners and Interaction Partner Closeness

By combining information on with whom participants interacted with, together with an item on interaction partner closeness, which was assessed after the EMA phase in a personal social network survey (see OSF for details), we derived the number of interaction partners and an interaction-partner closeness value per social interaction. The interaction partner closeness item asked “How close are you with this person?” on a 5-point Likert scale (0 = “really not close”, 4 = “very close”). When multiple interaction partners were reported (in *T* = 360 observations, 27% cases), a mean closeness score across all interaction partners present during the interaction was computed. Participants reported interacting on average with 1.50 (*SD* = 1.05) individuals per interaction report. The majority of interactions (*T* = 1,005, 71.4%) were reported to be with one interaction partner. In 201 (15%) of the observations, this variable was missing, due to interactions with strangers that were not rated in the post EMA-survey. Mean interaction partner closeness was 3.25 (*SD* = 0.92) across all interaction reports.

### Analytical Strategy

Analyses were preregistered (see OSF) and deviations thereof are documented in Table S1 of the Supplementary Materials. All main analyses were conducted in a multilevel SEM framework (Preacher et al., 2010), where observations *j* (Level 1) are nested within participants *i* (Level 2). We specified two multilevel mediation models using the R software *lavaan* (R version 4.3.1, lavaan version 0.6–16; Rosseel, 2012); one for the Spanish sample and one for the Dutch sample. The *lavaan* software automatically partitions the within- and the between-person variance and allows estimating parameters on the within- and between-person level. In the Spanish sample model, affect and loneliness constitute the two outcome variables. In the Dutch sample model, positive affect and negative affect enter the model as the main outcome variables. Given that we are mainly interested in the difference between FtF interactions and digital interactions, the $mode_{ij}$ variable only consists of two levels, with FtF interactions representing the reference category (i.e., a value of zero) and all digital interaction jointly as a value of one. The interaction quality items, as described above, were used to estimate a latent interaction quality variable $Qual_{ij}$ . For the Spanish sample, the single-item momentary well-being measures (i.e., affect, loneliness) entered the model as manifest variables, whereas in the Dutch sample the multi-item scales for positive and negative affect were used to estimate latent variables for positive and negative affect.

Hypothesis 1 (H1) is tested with the within-person effect of interaction mode $mode_{ij}$ on interaction quality $Qual_{ij}$ . H2 is assessed with the within-person effect of interaction quality $Qual_{ij}$ on momentary well-being indicators (i.e., affect, loneliness, positive affect, negative affect). H3 is examined by evaluating the total effects of interaction mode $mode_{ij}$ on momentary well-being outcomes (i.e., the direct effect plus the indirect effect; MacKinnon, 2008). We use Monte Carlo Confidence Intervals (Preacher & Selig, 2012), to assess the presence of a significant indirect effect via the mediator of interaction quality, testing H4. On the between-person level, the model is specified as fully saturated to account for between-person relations between the variables.^4^

We assessed the SEM model’s adequacy using the Comparative Fit Index (CFI; Bentler, 1990), the Root Mean Square Error of Approximation (RMSEA; Steiger, 1990), and the Standardized Root Mean Square Residual (SRMR; Kline, 2011). A good fit is indicated by CFI exceeding .95 and when both RMSEA and SRMR are below .08 (Hu & Bentler, 1999).

We also report on the between-person effects in the Spanish sample, but not for the Dutch Sample, due to the small number of individuals (*N*_2_ = 21) and thus low statistical power on the between-person level.

### Preregistered Robustness Analysis

For a mediation to be conceptually valid, we need to make sure that the predictor (X; interaction mode) temporally precedes the mediator (M; interaction quality) and the outcome (Y; momentary well-being) and that the mediator also precedes the outcome (MacKinnon, 2008). In our case, the type of interaction mode is determined at the start of an interaction and thus temporally precedes how individuals perceive the quality of the interaction. Similarly, the quality of the social interaction (i.e., the mediator) temporally precedes how participants feel after the social interaction. To further strengthen the argument about the temporal order of these processes, we additionally report a preregistered robustness analysis controlling for the previous time point in momentary well-being (i.e., affect, loneliness) in the Spanish sample.^5^ By controlling for previous momentary well-being, we can rule out the explanation that previous emotional states explained the mediation effect.

### Addition Preregistered Analyses

In a first preregistered additional analysis, we estimate a model in which we control for the number of interaction partners and interaction-partner closeness, as they may affect interaction quality and subsequent well-being (Bayer et al., 2021; Stadel, Van Duijn, Wright, et al., 2024).

In a second preregistered additional analysis, we explored whether it may also be that interaction quality does not act as a mediator, but rather that interaction mode moderates the relationship between interaction quality and momentary well-being. Following the analyses by Achterhof et al. (2022), who examined this moderation hypothesis in a sample of adolescents, we also estimated multilevel moderation SEMs, with interaction mode as a moderator. For this, we estimated the within-person effect of the interaction term of the manifest interaction mode and interaction quality variables on momentary well-being outcomes.

## Results

Before we turn to discussing the multilevel SEM results in light of our hypotheses, we describe the data in more detail and provide bivariate correlation analyses. The data of the Spanish sample, all R-Code documenting all reported analyses, more descriptive statistics, and detailed model results can be found on OSF. Data for the Dutch sample cannot be shared due to agreements with the ethics commission.

### Descriptive Statistics

The compliance rate of the signal-contingent reports was 72% in the Spanish sample and 82.7% in the Dutch sample. Additional 641 reports of event-contingent social interaction were reported in the Dutch sample. The total sample of reported interactions was 5,116 in the Spanish sample and 1,386 in the Dutch sample. Table 1 displays the mean values of pertinent time-varying variables categorized by sample. In the Spanish sample, we observed 3,330 FtF interactions (65%), 226 video calls (4%), 334 voice calls (7%), 1,036 texting interactions (20%), and 190 social media interactions (4%). In the Dutch sample, there were 1,067 FtF interactions (77%), 188 video calls (14%), and 131 voice calls (9%).

**Table 1.** Means (SD) of Time-Varying Variables by Sample.

Variable	Spanish sample Mean (*SD*)	Dutch sample Mean (*SD*)
Percentage digital interactions (interaction mode)^a^	0.36 (0.22)	0.24 (0.21)
Mean interaction quality^b^	4.98 (0.70)	7.94 (0.93)
Affect valence	4.77 (0.66)	-
Loneliness	2.74 (0.85)	-
Positive affect	-	6.57 (1.11)
Negative affect	-	2.21 (1.42)
Interaction partner closeness	-	3.25 (0.92)

*Note. N*_1_ = 216, *N*_2_ = 22 participants, *T*_1_ = 5,116, and *T*_2_ = 1,386 time points.

a *T*-test indicates significant differences between the samples in interaction mode (digital vs. FtF interactions), *t* (2,334) = 9.06, *p* < .001).

b No comparison between samples made due to differences in the scale levels between the samples.

**Figure 2.**
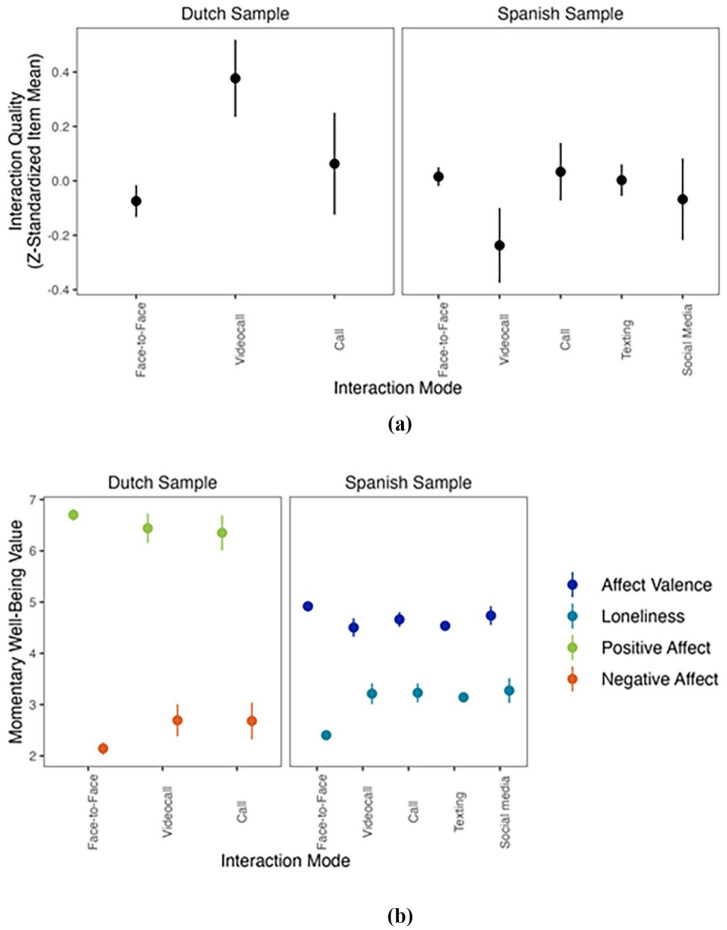
Mean and 95% confidence intervals for (a) Interaction quality by interaction mode and (b) Momentary well-being measures by interaction mode. *Note. N*_1_ = 216 participants, *T*_1_ = 5,116 time points. *N*_2_ = 22 participants, *T*_2_ = 1,386 time points. Some 95% Confidence Intervals are so small that point estimates (seem to) appear without confidence intervals. Interaction quality was z-standardized in Figure 2a to make the samples more comparable.

Figure 2 shows the means and 95% Confidence Intervals of quality and momentary well-being ratings per interaction mode. In Figure 2a, we can see that video calls were rated systematically lower than other modes of interactions in the Spanish sample but systematically higher in the Dutch sample.

Tables 2 and 3 show the within- and between-person correlations of interaction mode, interaction quality, and momentary well-being measures of the Spanish and Dutch samples, respectively. Tables 2 and 3 indicate that although in the Spanish sample there are no differences in interaction quality between the two interaction modes, in the Dutch sample digital interactions were rated higher in quality than FtF interactions. In both samples, interaction quality is associated with momentary well-being measures on a within-person level, and in the Spanish sample also on a between-person level. FtF interactions are associated with higher momentary well-being measures than digital interactions on a between- and within-person level in the Spanish sample, but not in the Dutch one.

**Table 2.** Within- and Between-Person Correlations in the Spanish Sample.

	Interaction mode^a^	Interaction quality	Affect valence	Loneliness
Interaction mode^a^	-	.00	−.12***	.23***
Interaction quality	−.03	-	.30***	−.24***
Affect valence	−.14*	.63***	-	−.36***
Loneliness	.28***	−.27***	-.35***	-

*Note. N*_1_ = 216 participants, *T*_1_ = 5,116 time points. Bottom triangle coefficients represent between-person correlations, and top triangle coefficients represent within-person correlations.

a Binary variable with reference category FtF, correlation coefficients represent Spearman rank correlations (otherwise Pearson correlations).

* *p* < .05. **p < .01. ***p < .001.

**Table 3.** Within- and Between-Person Correlations in the Dutch Sample.

Variable	Interaction mode^a^	Interaction quality	Positive affect	Negative affect
Interaction Mode^a^	-	.14***	.02	.01
Interaction quality	.19	-	.38***	−.25***
Positive affect	−.31	.37	-	−.59***
Negative affect	.38	−.18	−.63***	-

*Note. N*_2_ = 22 participants, *T*_2_ = 1386 time points. Bottom triangle coefficients represent between-person correlations, and top triangle coefficients represent within-person correlations.

a Binary variable with reference category FtF, correlation coefficients represent Spearman rank correlations (otherwise Pearson correlations).

* *p* < .05. **p < .01. ***p < .001.

### Multilevel Structural Equation Models

**Figure 3.**
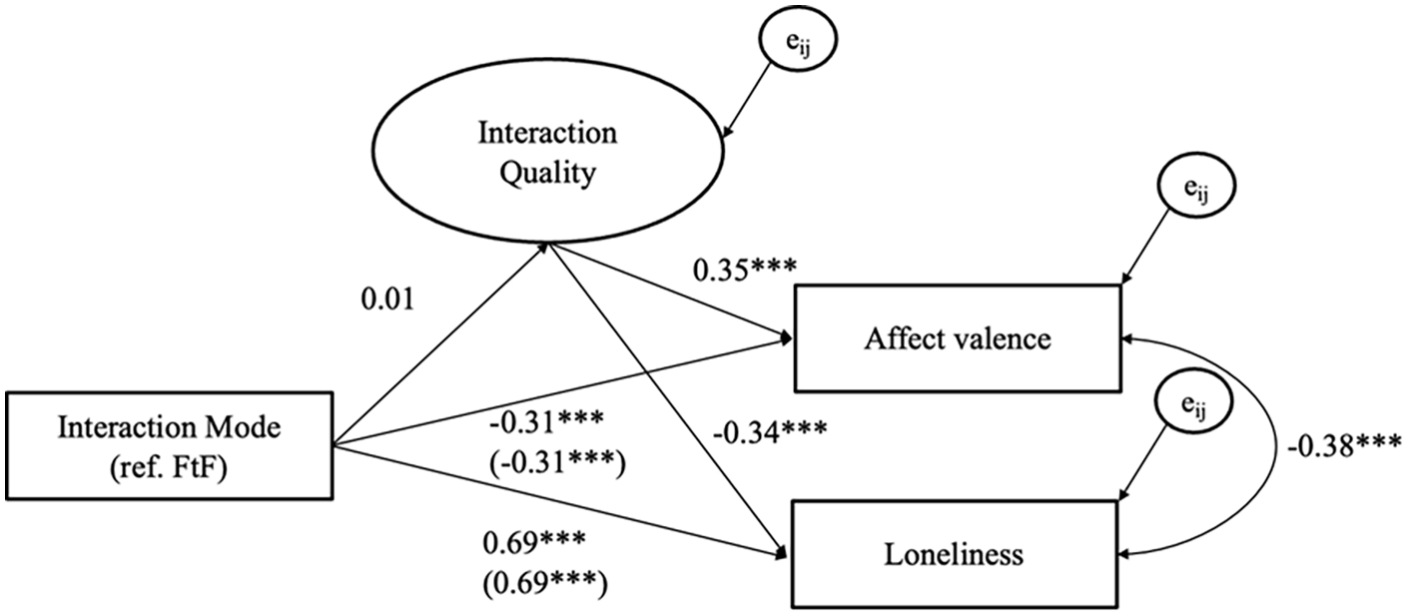
Within-person level unstandardized fixed effects coefficients for the Spanish sample. *Note. N_1_* = 216, *N_1obs_* = 5,116, ***p < .001, coefficients in brackets represent the total effects (i.e., without the mediator). e_ij_ represents the error terms of dependent variables. The model on the between-person level is fully saturated (i.e., estimated covariances between all manifest variables) but is not shown for readability. Age and gender were control variables. For more details, see Supplemental Table S2.

**Figure 4.**
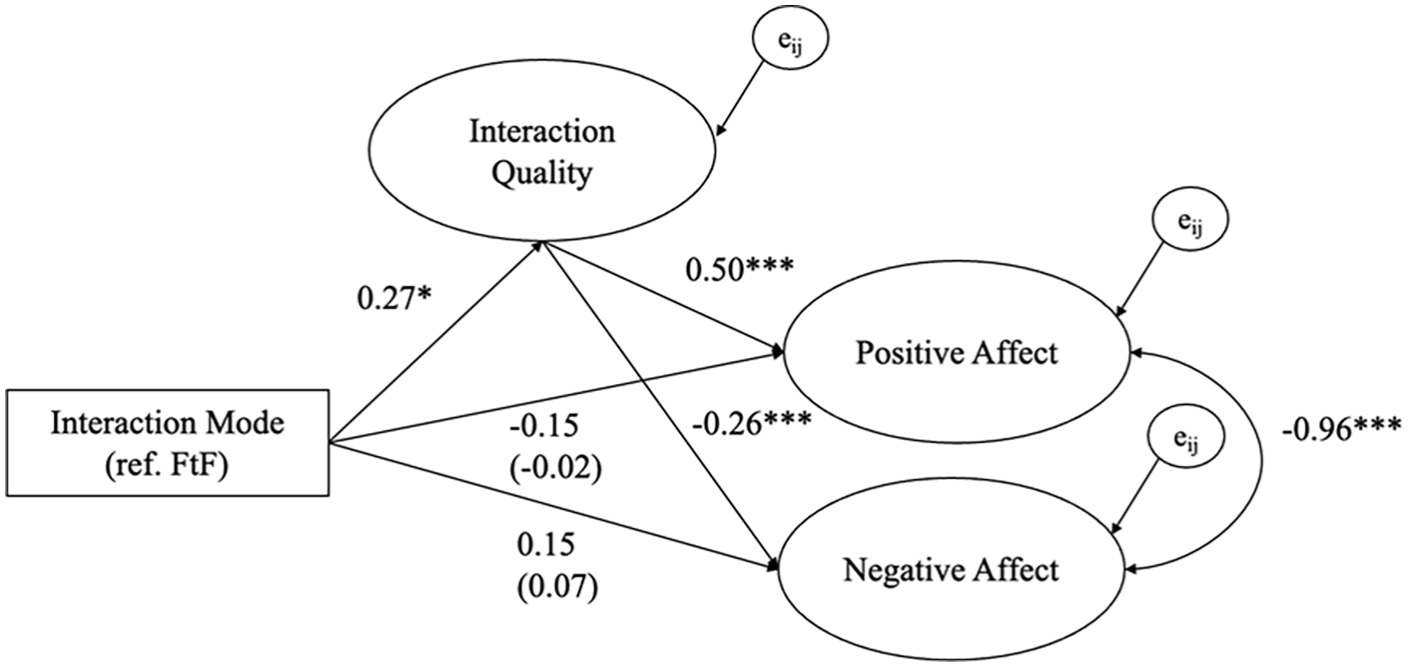
Within-person Level Unstandardized Fixed Effects Coefficients for the Dutch sample. *Note. N_2_* = 22, *N_2obs_* = 1,386, *p < .05, ***p < .001, coefficient in brackets represent the total effects (i.e., without the mediator). e_ij_ represents the error terms of dependent variables. The model on the between-person level is fully saturated (i.e., estimated covariances between all manifest variables) but is not shown for readability. Age and gender were control variables. For more details, see Supplemental Table S2.

Figures 3 and 4 show the estimated coefficients of the within-person multilevel SEM for the Spanish and Dutch samples, respectively. For detailed result tables of the models (including e.g., latent factor loadings), see Supplementary Materials Tables S2 and S3. Model fit in both models was good with CFI_S_ = .99, SRMR_S_ = .018, RMSEA_S_ = .038 for the Spanish sample and CFI_D_ = .95, SRMR_D_ = .038, RMSEA_D_ = .056 for the Dutch sample, indicating that the structure of the latent variables in the models align well with the data (Hu & Bentler, 1999).

#### H1. Digital interactions are associated with a lower level of perceived interaction quality than face-to-face interactions on a within-person level

The within-person multilevel SEM results, displayed in Figures 3 and 4, indicate that there were no within-person differences between digital and FtF interactions in the Spanish sample (*b* = 0.01, *SE* = 0.04, *p* = .842) and that in the Dutch sample digital interactions were rated higher in quality than FtF interactions (*b* = 0.27, *SE* = 0.11, *p* = .015). Although the effect was significant in the Dutch sample, its direction was contrary to our expectation. Hence, we did not find consistent support for H1. The explained variance of interaction quality on the within-person was *R*^2^_IS_ = .01 for the Spanish sample and *R*^2^_D_ = .01 for the Dutch sample.

#### H2. Interaction quality is positively associated with subsequent well-being on a within-person level

In the Spanish sample, interaction quality was positively associated with subsequent affect valence (*b* = 0.35, *SE* = 0.02, *p* < .001) and negatively associated with subsequent loneliness on a within-person level (*b* = −0.34, *SE* = 0.02, *p* < .001). In the Dutch sample, interaction quality was positively associated with subsequent positive affect (*b* = 0.50, *SE* = 0.03, *p* < .001) and negatively associated with subsequent negative affect on a within-person level (*b* = −0.26, *SE* = 0.03, *p* < .001). In both samples, we found support for the hypothesis that interaction quality is associated with subsequent well-being on a within-person level, wherein in moments when individuals report higher interaction quality than usual, they are more likely to experience higher levels of affect valence and positive affect as well as lower levels of loneliness and negative affect.

#### H3. Digital interactions are associated with a lower level of subsequent momentary well-being than face-to-face interactions on a within-person level

For this hypothesis, we report the total effect of interaction mode on momentary well-being measures, assuming no mediation in the model. Digital social interactions, compared to FtF interactions, were negatively associated on a within-person level with subsequent affect valence (*b* = −0.31, *SE* = 0.04, *p* < .001) and positively associated with loneliness in the Spanish sample (*b* = 0.69, *SE* = 0.04, *p* < .001). In the Dutch sample, there were no significant within-person associations between interaction mode and subsequent positive affect (*b* = −0.02, *SE* = 0.11, *p* = .860) or negative affect (*b* = 0.07, *SE* = 0.09, *p* = .429). Hence, we found support for H3 in the Spanish but did not in the Dutch sample.

#### H4. The within-person association between interaction mode and momentary well-being is mediated by interaction quality

In the Spanish sample, interaction quality did not mediate the relationship between interaction mode and subsequent well-being as the indirect effect was estimated as small and non-significant for affect valence (*b* = 0.00, *SE* = 0.01, *p* = .852) and loneliness (*b* = -0.00, *SE* = 0.01, *p* = .852). In the Dutch sample, the indirect effect was significant for positive affect (*b* = 0.13, *SE* = 0.06, *p* = .017) and negative affect (*b* = −0.07, *SE* = 0.03, *p* = .019). Hayes (2022) argues that the indirect effect (even in the absence of a main effect, like in our case) is informative of a mediation process. In sum, we found evidence for an indirect effect in the Dutch sample, but no evidence for interaction quality to mediate the relationship between interaction mode on momentary well-being on a within-person level in the Spanish sample. The explained variance of affect valence and loneliness on the within-person were *R*^2^_A_ = .16 and *R*^2^_L_ = .14 in the Spanish sample. In the Dutch sample, *R*^2^_PA_ = .33 and *R*^2^_NA_ = .14 of the variance on a within-person level were explained.

### (Preregistered) Robustness Analyses

We performed several robustness analyses, detailed in the Supplementary Materials (section Robustness Analyses). Here, we only briefly summarize the results of these analyses. First, re-estimating the Spanish sample model as preregistered, controlling for lag-1 effects on current well-being and interaction quality, yielded results similar in size and significance. Second, re-estimating our multilevel mediation SEM with manifest variables showed similar effect sizes and significance levels than reported above. Only in the Dutch sample, the negative effect of interaction mode on interaction quality increased in size and significance. Third, multilevel regression models with random slope terms for focal within-person effects provided comparable results, except for a non-significant within-person effect of interaction mode on interaction quality in the Dutch sample. Fourth, re-estimating the Spanish sample model with only FtF, calls, and video calls–matching the available categories in the Dutch sample–showed similar effect sizes and significance levels as reported above. Overall, these analyses support the robustness of our main findings across various modeling strategies and with the inclusion of relevant covariates.

### Additional Preregistered Analyses

#### Role of Interaction Partner Closeness and the Number of Interaction Partners

In the Dutch sample, we also examined the role of interaction partner closeness and the number of interaction partners (see Supplemental Table S7 for detailed results). Interestingly, closeness was associated with less positive affect (*b* = −0.12, *SE* = 0.06, *p* = .041). The number of interaction partners in a specific interaction was not associated with positive affect (*b* = 0.01, *SE* = 0.04, *p* = .848). Closeness and the number of interaction partners were not associated with subsequent negative affect (*b_closeness_* = 0.08, *SE* = 0.05, *p* = .151; *b_nPartner_* = -0.03, *SE* = 0.04, *p* = .469). Closeness, but not the number of interaction partners, was associated with higher interaction quality (*b_closeness_* = 0.35, *SE* = 0.06, *p* < .001; *b_nPartner_* = -0.05, *SE* = 0.04, *p* = .252). The digital modes of interaction were associated with higher interaction partner closeness (*b* = 0.48, *SE* = 0.06, *p* < .001) and fewer number of interaction partners (*b* = −.31, *SE* = 0.07, *p* < .001) compared to FtF interactions, indicating that digital interactions were more likely to take place with close interaction partners and in smaller groups than FtF interactions. When controlling for interaction partner closeness and the number of interaction partners the effect sizes of our focal coefficients became smaller and we observed higher p-values of our hypothesized effects, with the effect of interaction mode on interaction quality becoming non-significant (*b* = 0.13, *SE* = 0.12, *p* = .284), as well as the indirect effect in the Dutch sample (*b_PA_* = 0.07, *SE* = 0.06, *p* = .285; *b_PA_* = −0.04, *SE* = 0.04, *p* = .288).

#### Interaction Mode as a Moderating Variable

**Figure 5.**
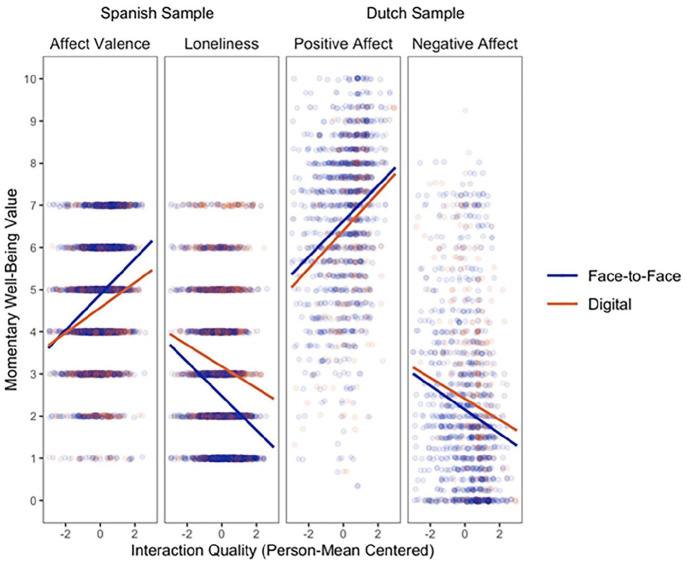
Model-implied moderation effect (and Raw Data) of interaction mode and interaction quality on momentary well-being measures. *Note. N_I_* = 216, *T_I_* = 5,116, *N_I_* = 22, *T_I_* = 1,386. Fitted lines are model-implied, (jittered) data points are raw data. Data on the x-axis represent the person-mean centered values of interaction quality, thus representing a within-person variable. Interaction quality moderates the within-person relationship between interaction mode and momentary well-being in the Spanish sample (affect valence: *b* = −0.09, *SE* = 0.03, *p* = .003, loneliness: *b* = 0.13, *SE* = 0.04, *p* < .001), but not in the Dutch sample (positive affect: *b* = 0.07, *SE* = 0.06, *p* = .258, negative affect: *b* = −0.02, *SE* = 0.06, *p* = .721).

We also examined whether interaction mode moderates the relationship between interaction mode and momentary well-being. A similar investigation was undertaken by Achterhof et al. (2022), although they did not find supporting evidence. It is noteworthy that their approach involved considering interaction quality as a person-level variable, differing from our method, where we assessed it at the within interactions level. To investigate the moderating effect of interaction mode, we estimated multilevel SEMs. Because the R-software lavaan cannot estimate models with interaction terms of latent variables, we used manifest mean variables of the predictor (i.e., mean of interaction quality items, both samples) and outcome variables (i.e., mean of positive or negative affect items; Dutch sample). The results of these models are reported in the Supplementary Materials Tables S8 and S9. These models suggest that interaction mode significantly moderates the within-person relationship between interaction quality and momentary well-being in the Spanish sample (affect valence: *b* = -0.09, *SE* = 0.03, *p* = .003, loneliness: *b* = 0.13, *SE* = 0.04, *p* < .001), but not in the Dutch sample (positive affect: *b* = 0.07, *SE* = 0.06, *p* = .258, negative affect: *b* = −0.02, *SE* = 0.06, *p* = .721). Figure 5 shows the raw data points and the model-implied fitted lines for this moderation effect. It displays that when individuals in the Spanish sample interact digitally, the within-person effect of interaction quality on momentary well-being is weaker than when interacting FtF. We further explored which interaction modes specifically were driving the moderation effect in the Spanish sample, and discussed this in the Supplementary Materials.

## Discussion

In this study, we investigated the interplay between interaction mode, interaction quality, and momentary well-being in two samples of individuals reporting on their social interactions in daily life with Ecological Momentary Assessments (EMAs). We examined four preregistered hypotheses. We did not find empirical support on a within-person level for Hypothesis 1, which predicted that digital social interactions are rated lower in their interaction quality than FtF interactions. Interaction quality was robustly associated with subsequent momentary well-being on a within-person level, supporting H2. Digital social interactions were associated with lower levels of subsequent momentary well-being in the Spanish sample but not in the Dutch sample, partially supporting H3. Finally, interaction quality mediated the relationship between interaction mode and momentary well-being in the Dutch but not in the Spanish sample, showing mixed support for H4. In preregistered robustness analysis, in which we controlled for interaction partner closeness and the number of interaction partners in the Dutch sample resulted in non-significant effects for the association between interaction mode and quality (H1) and the indirect effect of interaction quality (H4). In the preregistered exploratory analyses, we found evidence for a moderation effect of interaction mode with interaction quality on subsequent well-being in the Spanish but not in the Dutch sample. Below, we discuss (a) the results of each hypothesis in more detail and the implications for theory development, (b) reflections on conceptualizing and measuring interaction modality and quality, and (c) limitations and avenues for future research.

### Relationship Between Mode of Interaction and Interaction Quality (H1)

Contrary to our hypothesis and prior empirical evidence (Hall, Pennington, Merolla, 2023), we did not find that digital interactions were associated with lower levels of perceived interaction quality than FtF interactions on a within-person level. Indeed, the opposite effect was found in the Dutch sample; digital interactions were rated higher in quality than FtF interactions, which would align with some propositions of cues-filtered-in theories (Walther & Parks, 2002). Yet, this effect became insignificant when controlling for interaction partner closeness and the number of interaction partners present during the interaction. These null- and counter-direction findings of H1 align to some extent to the findings of Hülür et al. (2023) who showed that FtF interactions were only rated higher than phone calls with regard to meaningfulness, but not in other quality indicators measured (i.e., valence, social relatedness, calmness), and Hall, Pennington, and Holmstrom (2023) who found that voice calls showed similarly high values as FtF interactions in satisfying the need to belong. These findings suggest that the quality of digital and FtF interactions may not simply depend on the presence or absence of (nonverbal) cues but rather on individuals’ ability to adapt to the available cues based on situational and relational factors. This aligns with cues-filtered-in theories, which argue that people can effectively adapt to the limitations of mediated channels by leveraging contextual information to sustain quality interactions.

When examining various modes of interaction specifically (see Figure 2a), video calls were perceived as lower in quality in the Spanish sample and higher in quality in the Dutch sample compared to other interaction modes. This discrepancy suggests that video calls may hold a unique meaning to each sample of participants. Current theoretical frameworks do not provide explanations on (a) why video calls would be rated specifically lower or higher in quality than other modalities, and (b) why such differences between samples are observed. One possibility is that the effect of interaction mode on interaction quality is not uniform but instead depends on contextual factors such as the interaction partner, the location of the interaction, and its purpose (Fernández, Elmer, Sádaba, et al., 2025). We further speculate about conceptual- and measurement-related factors contributing to the inconsistent findings between the samples in a separate subsection further below.

### Relationship Between Social Interaction Quality and Momentary Well-Being (H2)

We found strong and robust support for the within-person relationship between interaction quality and subsequent momentary well-being (H2). In moments where participants experienced higher interaction quality than usual, they subsequently reported higher levels of momentary well-being. These findings resonate with prior research examining FtF interactions exclusively, underscoring the significance of interaction quality in shaping well-being outcomes (Bernstein et al., 2018; Berry & Hansen, 1996; Elmer, Ram, Gloster, et al., 2023; Hall, Holmstrom, Pennington, et al., 2023; Sun et al., 2019; Vogel et al., 2017). Our study extends this understanding by incorporating digital interactions and distinguishing the relationship between interaction quality and momentary well-being across different interaction modes (see Supplemental Figure S1).

Moreover, our contribution to the existing literature lies in demonstrating a robust association between social interaction quality and momentary well-being at the within-person level, encompassing two distinct operationalizations of interaction quality. As highlighted by Liu et al. (2019), there is no consensus in the literature on how social interaction quality can be operationalized. Consequently, research that disentangles the various dimensions of interaction quality from resulting affective experiences is needed. Our study addresses this gap, offering a top-down theory-driven approach in the Spanish sample and a bottom-up participant-driven approach in the Dutch sample to identify the dimensions of interaction quality.

### Relationship Between Mode of Interaction and Momentary Well-Being (H3)

We find support for the within-person effect of interaction mode on momentary well-being in the Spanish, but not in the Dutch sample. In other words, digital social interactions were associated with subsequent lower levels of affect valence and loneliness compared to FtF interactions, but no significant differences observed in positive and negative affect. The results of the Spanish sample align well with those of existing studies (Achterhof et al., 2022; Kafetsios et al., 2017; Kroencke et al., 2023; Kushlev & Heintzelman, 2018; Roshanaei et al., 2023).

Upon examining this association by the specific modes of interactions in exploratory analysis, we observed that the association between interaction quality and momentary well-being was higher after FtF, calls, and texting interactions, but not necessarily following video calls and social media interactions (see Supplemental Figure S1). Social media interactions pose challenges in conceptual clarification (Hall, 2018b) and we speculate on the findings of video chat below. Taken together, for the most frequently used modes of communication (i.e., FtF, text, phone calls) the association between quality and well-being is consistently positive and robust.

### Interplay Between Interaction Mode, Quality, and Momentary Well-Being (H4)

We found an indirect effect of interaction mode on momentary well-being via interaction quality in the Dutch, but not in the Spanish sample. However, when controlling for the number of interaction partners and interaction partner closeness in a robustness analysis, the indirect effect in the Dutch sample disappeared. In the Spanish sample, the absence of a mediation effect is likely attributed to the absence of an association between interaction mode and interaction quality (H1).

One potential reason for the absence of a mediation effect is that the dichotomy between digital and FtF interactions might oversimplify the diverse modes and their relationships with interaction quality and momentary well-being. In our study, we thus not only provide results for a binary measure of interaction mode (digital vs. FtF) in the main part of the manuscript, but also supplementary analyses for the Spanish sample (see Supplemental Table S10) in which distinguishing between specific modes of interactions. The overall conclusions drawn from the mode-specific models are identical to the ones from a binary-mode model.

Another potential reason for the absence of a mediating effect is the lack of control for contextual factors, for example, interaction purpose or content of communication (Goldsmith & Baxter, 1996; Hall, 2018a). For instance, a dry business meeting might receive a low-quality rating because its purpose is information provision rather than emotional social support. In this context, Hülür et al. (2023) found that interaction purpose only moderated the relationship between interaction mode and quality for specific combinations of modes and purposes (e.g., instrumental purpose, social support). Moreover, Fernández, Elmer, Sádaba, et al. (2025) recently found that location, purpose, and partner familiarity moderated the association between interaction mode and quality. For example, video calls were associated with higher quality than face-to-face interactions when at home, but not outside of one’s home (Fernández, Elmer, Sádaba, et al., 2025).

Although we did not control for such contextual factors, the inclusion of a *relational* factor–interaction partner closeness—provided further insights. Our additional preregistered analyses of the Dutch sample indicate that interactions with closer partners were rated higher in quality than interactions with less close partners (see Supplemental Table S7). When controlling for interaction partner closeness, we further found that the effect of interaction mode on quality became insignificant compared to the main model, and, consequently, so did the indirect (mediated) effect. Hence, interaction partner closeness might work as an important confounding variable, being associated with both the mode one is interacting with and the quality one experiences (Yang et al., 2014). Individuals are more likely to use certain modalities of communication with certain types of relationship partners. The change in our results when partner closeness is accounted for suggests that it may not be a difference between modalities that past work is capturing, but a difference in who people typically communicate with on that modality. This explanation plus Hülür et al. (2023) findings regarding communication purpose suggests that modality, partner, and communication content all play a role in explaining differences in quality and well-being.

The findings from our preregistered exploratory analyses help illustrate this possibility. We observed a significant moderation effect of interaction mode on the relationship between interaction quality and momentary well-being in the Spanish sample. In the Dutch sample, these effects were not significant. When the quality of a digital interaction is high, its positive effect on momentary well-being appears to be stronger in FtF interactions, compared to digital interactions. It is well-established that certain purposes of communication are of higher quality (Hall, 2020b). If people choose to engage in more supportive communication in their FtF interactions and more planning or instrumental talk in their digital communication (e.g., coordination and planning via text), the association between quality and well-being may be stronger for FtF communication.

To better unpack the complex interplay between modality, communication content, and emotional closeness with partners both confirmatory and experimental research are needed to draw more robust conclusions. This investigation suggests that simple distinctions (e.g., FtF are of higher quality than digital) are not warranted unless these other factors are accounted for in study design.

### Factors Contributing to Inconsistent Findings Across Samples

The two samples yielded different results for the hypotheses H1 and H3, indicating that our analyses do not provide conclusive evidence regarding the association between interaction mode and interaction quality (H1) and subsequent well-being (H3). These inconsistencies underscore the complexity of the constructs under investigation, particularly interaction mode and interaction quality, and suggest that our findings may be context-dependent (Fernández, Elmer, Sádaba, et al., 2025; Schnauber-Stockmann et al., 2025). In this subsection, we discuss how our conceptualization and measurement of interaction quality, sample differences, COVID context, and method may have contributed to these inconsistent results.

Although one strength of our analyses is that we used different samples and different operationalization of interaction quality, the observed inconsistencies in H1 results between the samples does not allow us to draw conclusions. Thus, future research is needed examining the conceptualization of interaction quality, its measurement, and its association with interaction mode.

At the same time, our inconsistent findings regarding interaction quality might have to do with specific differences between our samples. Specifically, that video calls were rated lower in quality in the Spanish sample but higher in quality in the Dutch sample. Three characteristics of our data may have contributed to this. First, the Spanish sample consisted of Spanish young adults that were mostly non-students and many of whom were working, whereas the Dutch sample consisted of predominantly international students in the first year of their studies,^6^ who possibly maintain their closest social relations through digital media and video calls (Yang et al., 2014), and thus, these interactions are rated higher in quality items on, for example, enjoyment, meaningfulness, and being oneself. This speculation is in line with our finding that digital interactions were more likely to take place with close interaction partners than FtF interactions and that once closeness was accounted for, differences disappeared.

Second, the data for the Dutch sample were collected during fall 2021 when there were COVID-related governmental measures in place (e.g., no sports classes were allowed and events had to end at 20:00 latest, restricting FtF contact and in-person university teaching; for details, see RIMV). A stringency index of COVID measures rated the period of the data collection in the Netherlands with an average of 41.88 (ranging from 35.69 to 50.37) on a scale from 0 to 100 (Hale et al., 2021). Thus, the participants in the Dutch sample possibly used digital modes of interaction during the COVID-19 pandemic for important, meaningful social interactions. This compliments the argument that video calls were reserved for close relationship partners by international students. Conversely, the data of the Spanish were collected in the spring of 2022, when there were fewer COVID-related restrictions in place in Spain (with an average of 35.39 on the COVID stringency index). Although it remains unclear how exactly these COVID-related restrictions affected communication in our samples specifically, it is apparent that a lot of the social life moved to the digital world with the outbreak of the pandemic (Elmer, Mepham, Stadtfeld, 2020; Hall et al., 2021).

Given these differences between the samples, we cannot draw conclusions on why differences in results emerged—we can only state how important contextual factors (such as possibly COVID restrictions) and relational factors (such as interaction partner closeness) are for the interpretation of results. Moreover, it is plausible that there are cultural differences between the two samples that required further investigation. Future research could investigate in more detail why the mode of video calls is differently used across and within individuals, and how this potentially relates to interaction quality and subsequent well-being differently than other modes of interactions.

### Limitations

Certain limitations of this research should be acknowledged. First, the two samples were based mainly on females (84% and 77%), which may limit the generalizability of our findings to more gender-diverse populations. However, there is currently limited evidence for gender-based moderation in the context of computer-mediated communication (Meier & Reinecke, 2021). Nonetheless, future studies with more gender-balanced samples are needed to examine the robustness of these findings.

Second, whilst we had an ample number of interactions to examine in the Dutch sample (*T*_2_ = 1386), the participant count was relatively small (*N*_2_ = 22). Our focus on within-person differences means that the statistical power of our analyses relies more on the number of interaction reports than the number of individuals (Snijders & Bosker, 1999). Additionally, the substantial difference in the quantity of analyzed social interactions between the two samples might have affected the study’s conclusions, given that the Spanish sample (*T*_1_ = 5,116) had nearly four times the number of interaction reports compared to the Dutch sample. Consequently, the statistical power was higher in the Spanish than in the Dutch Sample.

Third, in the Dutch sample, we did not measure texting and social media interactions, thus limiting the comparability between the samples and the generalizability of our findings of the Dutch sample to diverse forms of digital social interactions. The different digital interaction modes present in the data did not substantially affect the conclusions of our results. As shown in the analyses reported in Supplementary Materials Table S6, when the digital interactions in the Spanish sample were reduced to match the categories in the Dutch sample (i.e., video calls and calls), the results remained consistent.

Fourth, although our study employed a carefully defined order for our variables reflecting their conceptual sequence (interaction mode → interaction quality → momentary well-being), it is important to acknowledge that all measures were collected at a single time point. This cross-sectional design limits our ability to establish causal relationships, particularly within the mediation analysis. Although the temporal ordering is strong, it cannot rule out the possibility of reverse causation or omitted variables influencing the observed associations. Due to the inherent difficulty of measuring subjective experiences like interaction quality concurrently with the interaction itself, employing an experimental mediation design would be necessary to establish a higher level of causality (Pirlott & MacKinnon, 2016; Shrout & Bolger, 2002).

## Conclusion

The implications of social interaction mode on interaction quality and momentary well-being are complex, both theoretically and empirically. Although we found robust within-person associations between interaction quality and well-being, the association of interaction mode with quality and well-being may be more context-dependent, with situational, relational, and possibly pandemic-related factors being at play. The mixed support for our hypotheses highlights the need for future research to disentangle the interplay between interaction mode, interaction quality, and momentary well-being further, potentially by improving and streamlining conceptualizations and measurements of interaction modalities and interaction quality. Additionally, our findings call for a reconsideration of theories that primarily focus on the presence of social cues, advocating instead for models that additionally account for contextual and relational factors in shaping social interaction outcomes.

## Supplemental Material

sj-docx-1-crx-10.1177_00936502251341088 – Supplemental material for Day-to-day Social Interactions Online and Offline: The Interplay Between Interaction Mode, Interaction Quality, and Momentary Well-being Supplemental material, sj-docx-1-crx-10.1177_00936502251341088 for Day-to-day Social Interactions Online and Offline: The Interplay Between Interaction Mode, Interaction Quality, and Momentary Well-being by Timon Elmer, Aurelio Fernández, Jeffrey A. Hall and Marie Stadel in Communication Research

## Notes

1. Deviations from the preregistration are reported in Supplementary Materials Table S1.

2. These measures included: work from home, restrictions for entering public buildings for unvaccinated/untested individuals, early closing times of stores (20:00), restrictions on public events: maximum number of participants; end of events at 17:00, no sports classes, maximum group size if 75 people for educational events, maximally 4 visitors per day (see National Institute for Public Health and the Environment of the Netherlands; RIMV).

3. “[In focused social interactions] relational partners share a mutual focus of attention and engage in conversation. Focused social interaction requires that conversation partners recognize one another as unique individuals.” - (Hall, 2018b, p. 164)

4. It was not possible to jointly estimate a mediation model with latent on the between and within-person level due to negative estimated variances, a common issue in latent variable modeling (Kolenikov & Bollen, 2012). See Supplementary Table S4-S5 for a robustness analysis with manifest variables and a converged mediation model on both the within- and between-person level.

5. Due to the event-contingent sampling scheme, a time-lagged analysis was not possible in the Dutch sample as intervals between observations were not equally spaced and thus would violate assumptions of time-lagged analyses (Elmer, van Duijn, et al., 2023; Voelkle et al., 2012).

6. In the Dutch sample, a substantial 81% reported that their family resided outside the same country, and 76% indicated that their closest friends were also situated in different countries

## References

[bibr1-00936502251341088] Achterhof R. Kirtley O. J. Schneider M. Hagemann N. Hermans K. S. F. M. Hiekkaranta A. P. Lecei A. Lafit G. Myin-Germeys I. (2022). Adolescents’ real-time social and affective experiences of online and face-to-face interactions. Computers in Human Behavior, 129(December 2021), 107159. 10.1016/j.chb.2021.107159

[bibr2-00936502251341088] Bayer J. B. Triệu P. Ellison N. Schoenebeck S. Y. Falk E. B. (2021). Rejection sensitivity and interaction quality in everyday life. Journal of Social and Personal Relationships, 38(12), 3646–3668. 10.1177/02654075211034237

[bibr3-00936502251341088] Bentler P. (1990). Comparative fit indexes in structural models. Psychological Bulletin, 107(2), 238–246. 10.1037/0033-2909.107.2.238 2320703

[bibr4-00936502251341088] Bernstein M. J. Zawadzki M. J. Juth V. Benfield J. A. Smyth J. M. (2018). Social interactions in daily life: Within-person associations between momentary social experiences and psychological and physical health indicators. Journal of Social and Personal Relationships, 35(3), 372–394. 10.1177/0265407517691366

[bibr5-00936502251341088] Berry D. S. Hansen J. S. (1996). Positive affect, negative affect, and social interaction. Journal of Personality and Social Psychology, 71(4), 796–809. 10.1037/0022-3514.71.4.796

[bibr6-00936502251341088] Bolger N. Laurenceau J.P. (2013). Intensive longitudinal methods: An introduction to diary and experience sampling research. Guilford Press. https://www.guilford.com/books/Intensive-Longitudinal-Methods/Bolger-Laurenceau/9781462506781

[bibr7-00936502251341088] Brissette I. Cohen S. Seeman T. E. (2000). Measuring social integration and social networks. In Cohen S. Underwood L. Gotlieb B. (Eds.), Social support measurement and intervention: A guide for health and social scientists (pp. 53–85). Oxford University Press.

[bibr8-00936502251341088] Cohen S. (2004). Social relationships and health. The American Psychologist, 59(8), 676–684. 10.1037/0003-066X.59.8.676 15554821

[bibr9-00936502251341088] Cohen S. Ashby T. (1985). Stress, social support, and the buffering hypothesis. Psychological Bulletin, 98(2), 310–357. 3901065

[bibr10-00936502251341088] Cooke P. J. Melchert T. P. Connor K. (2016). Measuring well-being: A review of instruments. The Counseling Psychologist, 44(5), 730–757. 10.1177/0011000016633507

[bibr11-00936502251341088] Daft R. L. Lengel R. H. (1986). Organizational information requirements, media richness and structural design. Management Science, 32(5), 554–571. 10.1287/mnsc.32.5.554

[bibr12-00936502251341088] Deci E. L. Ryan R. M. (2012). Self-determination theory. In Handbook of theories of social psychology, Vol. 1 (pp. 416–436). Sage Publications Ltd. 10.4135/9781446249215.n21

[bibr13-00936502251341088] Diener E. (1984). Subjective well-being. Psychological Bulletin, 95(3), 542–575. 10.1037/0033-2909.95.3.542 6399758

[bibr14-00936502251341088] Duran R. L. Kelly L. (2017). Knapp’s model of relational development in the digital age. Iowa Journal of Communication, 49(1), 22. https://openurl.ebsco.com/contentitem/gcd:136797458?sid=ebsco:plink:crawler&id=ebsco:gcd:136797458

[bibr15-00936502251341088] Eden J. Veksler A. E. (2016). Relational maintenance in the digital age: Implicit rules and multiple modalities. Communication Quarterly, 64(2), 119–144. 10.1080/01463373.2015.1103279

[bibr16-00936502251341088] Elmer T. Geschwind N. Peeters F. Wichers M. Bringmann L. (2020). Getting stuck in social isolation: Solitude inertia and depressive symptoms. Journal of Abnormal Psychology, 129(7), 713–723. 10.1037/abn0000588 32672987

[bibr17-00936502251341088] Elmer T. Mepham K. Stadtfeld C. (2020). Students under lockdown: Comparisons of students’ social networks and mental health before and during the COVID-19 crisis in Switzerland. PLoS ONE, 15(7 July), 1–22. 10.1371/journal.pone.0236337 PMC7377438 32702065

[bibr18-00936502251341088] Elmer T. Ram N. Gloster A. T. Bringmann L. F. (2023). Studying daily social interaction quantity and quality in relation to depression change: A multi-phase experience sampling study. Personality and Social Psychology Bulletin, 1–16. 10.1177/01461672231211469 PMC12130595 38098172

[bibr19-00936502251341088] Elmer T. van Duijn M. A. J. Ram N. Bringmann L. F. (2023). Modeling categorical time-to-event data: The example of social interaction dynamics captured with event-contingent experience sampling methods. Psychological Methods, No Pagination Specified-No Pagination Specified. 10.1037/met0000598 37676164

[bibr20-00936502251341088] Fernández A. Elmer T. Sádaba C. García-Manglano J. Vanden Abeele M. (2025). The quality of face-to-face and digitally mediated social interactions: Two experience sampling studies exploring the moderating role of physical location, interaction partner familiarity, and interaction purpose. Journal of Computer-Mediated Communication, 30(2), zmaf004. 10.1093/jcmc/zmaf004

[bibr21-00936502251341088] Fernández A. Vanden Abeele M. Sádaba C. García-Manglano J. Weinstein N. (2025). Feeling valued as a conversation-specific relational experience: An examination of Buber’s existential dialogical theory. The Journal of Positive Psychology, 1–14. 10.1080/17439760.2025.2481046

[bibr22-00936502251341088] Finch J. F. Okun M. A. Pool G. J. Ruehlman L. S. (1999). A comparison of the influence of conflictual and supportive social interactions on psychological distress. Journal of Personality, 67(4), 581–621. 10.1111/1467-6494.00066 10444852

[bibr23-00936502251341088] Fox J. McEwan B. (2017). Distinguishing technologies for social interaction: The perceived social affordances of communication channels scale. Communication Monographs, 84(3), 298–318. 10.1080/03637751.2017.1332418

[bibr24-00936502251341088] Goldsmith D. J. Baxter L. A. (1996). Constituting relationships in talk a taxonomy of speech events in social and personal relationships. Human Communication Research, 23(1), 87–114. 10.1111/j.1468-2958.1996.tb00388.x

[bibr25-00936502251341088] Goodman-Deane J. Mieczakowski A. Johnson D. Goldhaber T. Clarkson P. J. (2016). The impact of communication technologies on life and relationship satisfaction. Computers in Human Behavior, 57, 219–229. 10.1016/j.chb.2015.11.053

[bibr26-00936502251341088] Hale T. Angrist N. Goldszmidt R. Kira B. Petherick A. Phillips T. Webster S. Cameron-Blake E. Hallas L. Majumdar S. Tatlow H. (2021). A global panel database of pandemic policies (Oxford COVID-19 Government Response Tracker). Nature Human Behaviour, 5(4), 4. 10.1038/s41562-021-01079-8 33686204

[bibr27-00936502251341088] Hall J. A. (2018a). Energy, episode, and relationship: A test of communicate bond belong theory. Communication Quarterly, 66(4), 380–402. 10.1080/01463373.2017.1411377

[bibr28-00936502251341088] Hall J. A. (2018b). When is social media use social interaction? Defining mediated social interaction. New Media & Society, 20(1), 162–179. 10.1177/1461444816660782

[bibr29-00936502251341088] Hall J. A. (Ed.). (2020a). Mode comparison and coexistence. In Relating Through Technology (pp. 91–111). Cambridge University Press. 10.1017/9781108629935.006

[bibr30-00936502251341088] Hall J. A. (2020b). Relating through technology. Cambridge University Press. 10.1017/9781108629935

[bibr31-00936502251341088] Hall J. A. Davis D. C. (2017). Proposing the communicate bond belong theory: evolutionary intersections with episodic interpersonal communication: Proposing the communicate bond belong theory. Communication Theory, 27(1), 21–47. 10.1111/comt.12106

[bibr32-00936502251341088] Hall J. A. Dominguez J. Mihailova T. (2023). Interpersonal media and face-to-face communication: Relationship with life satisfaction and loneliness. Journal of Happiness Studies, 24(1), 331–350. 10.1007/s10902-022-00581-8 36406047 PMC9647761

[bibr33-00936502251341088] Hall J. A. Holmstrom A. J. Pennington N. Perrault E. K. Totzkay D. (2023). Quality conversation can increase daily well-being. Communication Research, 3, 00936502221139363. 10.1177/00936502221139363

[bibr34-00936502251341088] Hall J. A. Merolla A. J. (2020). Connecting everyday talk and time alone to global well-being. Human Communication Research, 46(1), 86–111. 10.1093/hcr/hqz014

[bibr35-00936502251341088] Hall J. A. Pennington N. Holmstrom A. (2021). Connecting through technology during COVID-19. Human Communication & Technology, 3(1), 15026. 10.17161/hct.v3i1.15026

[bibr36-00936502251341088] Hall J. A. Pennington N. Merolla A. J. (2023). Which mediated social interactions satisfy the need to belong? Journal of Computer-Mediated Communication, 28(1), zmac026. 10.1093/jcmc/zmac026

[bibr37-00936502251341088] Hayes A. F. (2022). Introduction to mediation, moderation, and conditional process analysis: A regression-based approach, Third Edition. Guilford Press.

[bibr38-00936502251341088] Holt-Lunstad J. Smith T. B. Baker M. Harris T. Stephenson D. (2015). Loneliness and social isolation as risk factors for mortality: A meta-analytic review. Perspectives on Psychological Science, 10(2), 227–237. 10.1177/1745691614568352 25910392

[bibr39-00936502251341088] Holt-Lunstad J. Smith T. B. Layton J. B. (2010). Social relationships and mortality risk: A meta-analytic review. PLoS Medicine, 7(7), 316. 10.1371/journal.pmed.1000316 PMC2910600 20668659

[bibr40-00936502251341088] Hu L. Bentler P. M. (1999). Cutoff criteria for fit indexes in covariance structure analysis: Conventional criteria versus new alternatives. Structural Equation Modeling: A Multidisciplinary Journal, 6(1), 1–55. 10.1080/10705519909540118

[bibr41-00936502251341088] Hülür G. Luo M. Macdonald B. Grünjes C. E. (2023). The perceived quality of social interactions differs by modality and purpose: An event-contingent experience sampling study with older adults. Journal of Social and Personal Relationships, 41, 02654075231215269. 10.1177/02654075231215269

[bibr42-00936502251341088] Ilies R. Johnson M. D. Judge T. A. Keeney J. (2011). A within-individual study of interpersonal conflict as a work stressor: Dispositional and situational moderators. Journal of Organizational Behavior, 32(1), 44–64. 10.1002/job.677

[bibr43-00936502251341088] Ishii K. Lyons M. M. Carr S. A. (2019). Revisiting media richness theory for today and future. Human Behavior and Emerging Technologies, 1(2), 124–131. 10.1002/hbe2.138

[bibr44-00936502251341088] Kafetsios K. Chatzakou D. Tsigilis N. Vakali A. (2017). Experience of emotion in face to face and computer-mediated social interactions: An event sampling study. Computers in Human Behavior, 76, 287–293. 10.1016/j.chb.2017.07.033

[bibr45-00936502251341088] Kahneman D. Krueger A. B. Schkade D. A. Schwarz N. Stone A. A. (2004). A survey method for characterizing daily life experience: The day reconstruction method. Science, 306(5702), 1776–1780. 10.1126/science.1103572 15576620

[bibr46-00936502251341088] Kline R. B. (2011). Principles and practice of structural equation modeling. Guilford Publications.

[bibr47-00936502251341088] Kolenikov S. Bollen K. A. (2012). Testing Negative Error Variances: Is a Heywood Case a Symptom of Misspecification? Sociological Methods & Research, 41(1), 124–167. 10.1177/0049124112442138

[bibr48-00936502251341088] Kroencke L. Harari G. M. Back M. D. Wagner J. (2023). Well-being in social interactions: Examining personality-situation dynamics in face-to-face and computer-mediated communication. Journal of Personality and Social Psychology, 124(2), 437–460. 10.1037/pspp0000422 35834202

[bibr49-00936502251341088] Kushlev K. Heintzelman S. J. (2018). Put the phone down: Testing a complement-interfere model of computer-mediated communication in the context of Face-to-Face interactions. Social Psychological and Personality Science, 9(6), 702–710. 10.1177/1948550617722199

[bibr50-00936502251341088] Lakey B. Orehek E. (2011). Relational regulation theory: A new approach to explain the link between perceived social support and mental health. Psychological Review, 118(3), 482–495. 10.1037/a0023477 21534704

[bibr51-00936502251341088] Langener A. M. Bringmann L. F. Kas M. J. Stulp G. (2024). Predicting mood based on the social context measured through the experience sampling method, digital phenotyping, and social networks. Administration and Policy in Mental Health and Mental Health Services Research, 51(4), 455–475. 10.1007/s10488-023-01328-0 38200262 PMC11196304

[bibr52-00936502251341088] Langener A. M. Stulp G. Jacobson N. C. Costanzo A. Jagesar R. R. Kas M. J. Bringmann L. F. (2024). It’s all about timing: Exploring different temporal resolutions for analyzing digital-phenotyping data. Advances in Methods and Practices in Psychological Science, 7(1), 25152459231202676. 10.1177/25152459231202677

[bibr53-00936502251341088] Langener A. M. Stulp G. Kas M. J. Bringmann L. F. (2023). Capturing the dynamics of the social environment through experience sampling methods, passive sensing, and egocentric networks: Scoping review. JMIR Mental Health, 10(1), e42646. 10.2196/42646 PMC10132048 36930210

[bibr54-00936502251341088] Lin X. Y. Lachman M. E. (2021). Daily stress and affect across adulthood: The role of social interactions via different communication modes. Technology, Mind, and Behavior, 1(3), 26. 10.1037/tmb0000026 PMC8974319 35369392

[bibr55-00936502251341088] Liu H. Xie Q. W. Lou V. W. Q. (2019). Everyday social interactions and intra-individual variability in affect: A systematic review and meta-analysis of ecological momentary assessment studies. Motivation and Emotion, 43(2), 339–353. 10.1007/s11031-018-9735-x

[bibr56-00936502251341088] Macdonald B. Luo M. Hülür G. (2021). Daily social interactions and well-being in older adults: The role of interaction modality. Journal of Social and Personal Relationships, 38(12), 3566–3589. 10.1177/02654075211052536

[bibr57-00936502251341088] MacKinnon D. (2008). Introduction to statistical mediation analysis. Routledge.

[bibr58-00936502251341088] Meier A. Reinecke L. (2021). Computer-mediated communication, social media, and mental health: A conceptual and empirical meta-review. Communication Research, 48(8), 1182–1209. 10.1177/0093650220958224

[bibr59-00936502251341088] Okdie B. M. Guadagno R. E. Bernieri F. J. Geers A. L. Mclarney-Vesotski A. R. (2011). Getting to know you: Face-to-face versus online interactions. Computers in Human Behavior, 27(1), 153–159. 10.1016/j.chb.2010.07.017

[bibr60-00936502251341088] Pirlott A. G. MacKinnon D. P. (2016). Design approaches to experimental mediation. Journal of Experimental Social Psychology, 66, 29–38. 10.1016/j.jesp.2015.09.012 27570259 PMC4999253

[bibr61-00936502251341088] Preacher K. J. Selig J. P. (2012). Advantages of Monte Carlo confidence intervals for indirect effects. Communication Methods and Measures, 6(2), 77–98. 10.1080/19312458.2012.679848

[bibr62-00936502251341088] Preacher K. J. Zyphur M. J. Zhang Z. (2010). A general multilevel SEM framework for assessing multilevel mediation. Psychological Methods, 15(3), 209–233. 10.1037/a0020141 20822249

[bibr63-00936502251341088] Reis H. T. Wheeler L. (1991). Studying social interaction with the rochester interaction record. Advances in Experimental Social Psychology, 24(C), 269–318. 10.1016/S0065-2601(08)60332-9

[bibr64-00936502251341088] Rice R. E. Love G. (1987). Electronic emotion: Socioemotional content in a computer-mediated communication network. Communication Research, 14(1), 85–108. 10.1177/009365087014001005

[bibr65-00936502251341088] Rook K. S. (2001). Emotional health and positive versus negative social exchanges: A daily diary analysis. Applied Developmental Science, 5(2), 86–97. 10.1207/S1532480XADS0502_4

[bibr66-00936502251341088] Roshanaei M. Vaid S. S. Courtney A. L. Soh S. J. Zaki J. Harari G. M. (2023). Contextualizing meaningful social interactions and momentary well-being in everyday life [Preprint]. PsyArXiv. 10.31234/osf.io/gu4pv

[bibr67-00936502251341088] Rosseel Y. (2012). lavaan: An R package for structural equation modeling. Journal of Statistical Software, 48(2), 1–26.

[bibr68-00936502251341088] Ruppel E. K. Gross C. Stoll A. Peck B. S. Allen M. Kim S.-Y. (2017). Reflecting on connecting: Meta-analysis of differences between computer-mediated and face-to-face self-disclosure. Journal of Computer-Mediated Communication, 22(1), 18–34. 10.1111/jcc4.12179

[bibr69-00936502251341088] Ryan R. M. Deci E. L. (2000). Self-determination theory and the facilitation of intrinsic motivation, social development, and well-being. American Psychologist, 55(1), 68–78. 10.1037/0003-066X.55.1.68 11392867

[bibr70-00936502251341088] Schnauber-Stockmann A. Bayer J. B. Harari G. M. Karnowski V. (2025). The situation in media and communication research. Communication Theory, 35(1), 25–36. 10.1093/ct/qtae021

[bibr71-00936502251341088] Short J. Williams E. Christie B. (1976). The social psychology of telecommunications. Wiley. http://bvbr.bib-bvb.de:8991/F?func=service&doc_library=BVB01&local_base=BVB01&doc_number=002037413&line_number=0001&func_code=DB_RECORDS&service_type=MEDIA

[bibr72-00936502251341088] Shrout P. Bolger N. (2002). Mediation in experimental and nonexperimental studies: New procedures and recommendations. Psychological Methods, 7, 422–445. 10.1037/1082-989X.7.4.422 12530702

[bibr73-00936502251341088] Silva G. Rum R. Brennan J. Rottenberg J. Goodman F. R. (2023). What allays loneliness? A fine-grained examination of daily social interactions. Journal of Social and Personal Relationships, 40(11), 3585–3609. 10.1177/02654075231181709

[bibr74-00936502251341088] Snijders T. A. B. Bosker R. J. (1999). An introduction to basic and advanced multilevel modeling-sage publications Ltd (1999).pdf. In Book. 10.1136/bcr-2013-009959

[bibr75-00936502251341088] Stadel M. Bringmann L. F. Stulp G. Elmer T. Verdonck S. Mestdagh M. van Duijn M. A. J. (2024). Capturing the social life of a person by integrating experience-sampling methodology and personal-social-network assessments. Advances in Methods and Practices in Psychological Science, 7(2), 25152459241244744. 10.1177/25152459241244745

[bibr76-00936502251341088] Stadel M. Stulp G. Langener A. M. Elmer T. Van Duijn M. A. J. Bringmann L. F. (2023). Feedback about a person’s social context—Personal networks and daily social interactions. Administration and Policy in Mental Health and Mental Health Services Research, 51(4), 476–489. 10.1007/s10488-023-01293-8 37615808 PMC11196300

[bibr77-00936502251341088] Stadel M. Van Duijn M. A. J. Wright A. G. C. Bringmann L. F. Elmer T. (2024). Considering the ‘With Whom’: Differences between event- and signal-contingent ESM data of person-specific social interactions. Multivariate Behavioral Research, 59, 1–18. 10.1080/00273171.2024.2335405 38590231

[bibr78-00936502251341088] Steiger J. H. (1990). Structural model evaluation and modification: An interval estimation approach. Multivariate Behavioral Research, 25(2), 173–180. 10.1207/s15327906mbr2502_4 26794479

[bibr79-00936502251341088] Sun J. Harris K. Vazire S. (2019). Is well-being associated with the quantity and quality of social interactions? Journal of Personality and Social Psychology, 2019(August 2019), 272. 10.1037/pspp0000272 31647273

[bibr80-00936502251341088] Trepte S. Masur P. K. Scharkow M. (2018). Mutual friends’ social support and self-disclosure in face-to-face and instant messenger communication. The Journal of Social Psychology, 158(4), 430–445. 10.1080/00224545.2017.1398707 29099670

[bibr81-00936502251341088] Trull T. J. Ebner-Priemer U. W. (2014). The Role of Ambulatory Assessment in Psychological Science. Current Directions in Psychological Science, 23(6), 466–470. 10.1177/0963721414550706 25530686 PMC4269226

[bibr82-00936502251341088] Verster J. C. Sandalova E. Garssen J. Bruce G. (2021). The use of single-item ratings versus traditional multiple-item questionnaires to assess mood and health. European Journal of Investigation in Health, Psychology and Education, 11(1), 183–198. 10.3390/ejihpe11010015 34542458 PMC8314344

[bibr83-00936502251341088] Voelkle M. C. Oud J. H. L. Davidov E. Schmidt P. (2012). An SEM Approach to continuous time modeling of panel data: Relating authoritarianism and anomia. Psychological Methods, 17(2), 176–192. 10.1037/a0027543 22486576

[bibr84-00936502251341088] Vogel N. Ram N. Conroy D. E. Pincus A. L. Gerstorf D. (2017). How the social ecology and social situation shape individuals’ affect valence and arousal. Emotion, 17(3), 509–527. 10.1037/emo0000244 27869467

[bibr85-00936502251341088] Walther J. B. (1992). Interpersonal effects in computer-mediated interaction: A relational perspective. Communication Research, 19(1), 52–90. 10.1177/009365092019001003

[bibr86-00936502251341088] Walther J. B. (1996). Computer-mediated communication: Impersonal, interpersonal, and hyperpersonal interaction. Communication Research, 23(1), 3–43. 10.1177/009365096023001001

[bibr87-00936502251341088] Walther J. B. (2011). Visual cues in computer-mediated communication: Sometimes less is more. In Face-to-face communication over the internet: Emotions in a web of culture, language and technology (pp. 17–38). Cambridge University Press. 10.1017/CBO9780511977589.003

[bibr88-00936502251341088] Walther J. B. Parks M. R. (2002). Cues filtered out, cues filtered in: Computer-mediated communication and relationships. In Handbook of interpersonal communication. Thousand Oaks, CA: Sage. https://www.semanticscholar.org/paper/Cues-Filtered-Out%2C-Cues-Filtered-In%3A-Communication-Walther-Parks/86a7daa28d8075d9791781ddd24c7d1a7d49649a

[bibr89-00936502251341088] Wichers M. (2014). The dynamic nature of depression: A new micro-level perspective of mental disorder that meets current challenges. Psychological Medicine, 44(7), 1349–1360. 10.1017/S0033291713001979 23942140

[bibr90-00936502251341088] Yang C. Brown B. B. Braun M. T. (2014). From Facebook to cell calls: Layers of electronic intimacy in college students’ interpersonal relationships. New Media & Society, 16(1), 5–23. 10.1177/1461444812472486

