# Supplementary material for: Day-to-day Social Interactions Online and Offline: The Interplay Between Interaction Mode, Interaction Quality, and Momentary Well-being
Source: Communic Res. 2025 May 31;53(5):713–46. doi: 10.1177/00936502251341088 (PMC13235958; doi:10.1177/00936502251341088)
Supplement: sj-docx-1-crx-10.1177_00936502251341088 – Supplemental material for Day-to-day Social Interactions Online and Offline: The Interplay Between Interaction Mode, Interaction Quality, and Momentary Well-being [file sj-docx-1-crx-10.1177_00936502251341088.docx]

**Supplementary Materials**

**Day-to-day social interactions online and offline: The interplay between interaction mode, interaction quality, and momentary well-being**Timon Elmer^1*^, Aurelio Fernández^2,3,4*^, Jeffrey A. Hall^5^ & Marie Stadel^6,7^

^1^ University of Zurich, Department of Psychology, Zurich, Switzerland

^2^ Ghent University, imec-mict, Department of Communication Science, Ghent, Belgium

^3^ University of Navarra, Faculty of Communication, Pamplona, Spain

^4^ University of Navarra, Institute of Culture and Society, Pamplona, Spain

^5^ University of Kansas, Department of Communication Studies, Lawrence, KS, USA

^6^ University of Groningen, Faculty of Behavioural and Social Sciences, Department of Psychometrics and Statistics, Groningen, The Netherlands

^7^ University of Groningen, Faculty of Behavioural and Social Sciences, Department of Sociology, Groningen, The Netherlands

^*^ These authors contributed equally

Correspondence concerning this article should be addressed to Timon Elmer,

University of Zurich, Applied Social and Health Psychology, Binzmühlestrasse 14/ Box 14, 8050 Zurich, Switzerland. Email: timon.elmer@uzh.ch

**Table S1**

*Deviations from Preregistration*

| **Original Plan** | **Deviation** | **Reason** |
| --- | --- | --- |
| Separate multilevel SEM are estimated per outcome variable (e.g., affect, loneliness) | Per sample one multilevel SEM is estimated with both main outcome variables in the model | An advantage of estimating one model per dataset is that the covariance between outcome variables can be estimated too |
| Interaction quality, positive and negative affect are manifest variables in the SEM | We used latent variables whenever possible in the SEM framework | If the model fits well, latent variables can be modeled more reliabily than their manifest counterparts (Borsboom, 2008) |
| Using a third sample | Prior to seeing the data, we decided to drop the analysis for Sample III | We realized only after the preregistration that the data of Sample III was only consisting of a one-item measure of positive affect. Importantly, this item asked about how participants felt *during* the social interaction. This is an issue for two reasons: First, this shift in the conceptual order would not have allowed to test our hypotheses about *subsequent* well-being nor our mediation hypothesis, because the mediator does not temporally precede the outcome. Second, the analysis of Sample II and III could not be pooled for greater statistical power. |
|  |  |  |

**Table S2**

*Detailed Multilevel SEM Results of Sample I*

| **Level 1 (within-person)** | |  |  |  |  |
| --- | --- | --- | --- | --- | --- |
| *Latent Variables* | |  |  |  |  |
| Latent factor | manifest variables | Estimate | *SE* | *z* | *p* |
| interaction quality latent | |  |  |  |  |
|  | quality item 1 | 1.00 |  |  |  |
|  | quality item 2 | 0.81 | 0.02 | 54.10 | 0 |
|  | quality item 3 | 0.59 | 0.01 | 46.11 | 0 |
| *Regressions* |  |  |  |  |  |
| Dependent variable | Independent variable | Estimate | *SE* | *z* | *p* |
| affect |  |  |  |  |  |
|  | interaction mode (ref. FtF) | -0.31 | 0.04 | -8.94 | 0 |
|  | interaction quality latent | 0.35 | 0.02 | 21.39 | 0 |
| loneliness |  |  |  |  |  |
|  | interaction mode (ref. FtF) | 0.69 | 0.04 | 16.55 | 0 |
|  | interaction quality latent | -0.34 | 0.02 | -17.41 | 0 |
| interaction quality latent | |  |  |  |  |
|  | interaction mode (ref. FtF) | 0.01 | 0.04 | 0.20 | 0.842 |
| *Covariances* | |  |  |  |  |
|  |  | Estimate | *SE* | *z* | *p* |
| affect |  |  |  |  |  |
|  | loneliness | -0.38 | 0.02 | -18.42 | 0 |
| *Intercepts* |  |  |  |  |  |
|  |  | Estimate | SE | z | p |
|  | quality item 1 | 0.00 |  |  |  |
|  | quality item 2 | 0.00 |  |  |  |
|  | quality item 3 | 0.00 |  |  |  |
|  | affect | 0.00 |  |  |  |
|  | loneliness | 0.00 |  |  |  |
|  | interaction quality latent | 0.00 |  |  |  |
| *Variances* |  |  |  |  |  |
|  |  | Estimate | *SE* | *z* | *p* |
|  | quality item 1 | 0.30 | 0.02 | 18.46 | 0 |
|  | quality item 2 | 0.41 | 0.01 | 31.28 | 0 |
|  | quality item 3 | 0.47 | 0.01 | 42.23 | 0 |
|  | affect | 1.13 | 0.02 | 48.52 | 0 |
|  | loneliness | 1.62 | 0.03 | 48.87 | 0 |
|  | interaction quality latent | 1.07 | 0.03 | 34.46 | 0 |
| *R-Square* |  |  |  |  |  |
|  |  | Estimate |  |  |  |
|  | quality item 1 | 0.78 |  |  |  |
|  | quality item 2 | 0.64 |  |  |  |
|  | quality item 3 | 0.44 |  |  |  |
|  | affect | 0.12 |  |  |  |
|  | loneliness | 0.12 |  |  |  |
|  | interaction quality latent | 0.00 |  |  |  |
| **Level 2 (between-person)** | |  |  |  |  |
| *Covariances* | |  |  |  |  |
| Dependent variable | Independent variable | Estimate | *SE* | *z* | *p* |
| quality item 1 | |  |  |  |  |
|  | quality item 2 | 0.51 | 0.06 | 9.25 | 0 |
|  | quality item 3 | 0.35 | 0.04 | 8.05 | 0 |
|  | interaction mode (ref. FtF) | -0.02 | 0.01 | -1.71 | 0.088 |
|  | affect | 0.27 | 0.04 | 6.89 | 0 |
|  | loneliness | -0.18 | 0.05 | -3.88 | 0 |
| quality item 2 | |  |  |  |  |
|  | quality item 3 | 0.39 | 0.05 | 8.44 | 0 |
|  | interaction mode (ref. FtF) | -0.02 | 0.01 | -1.95 | 0.052 |
|  | affect | 0.30 | 0.04 | 7.22 | 0 |
|  | loneliness | -0.18 | 0.05 | -3.88 | 0 |
| quality item 3 | |  |  |  |  |
|  | interaction mode (ref. FtF) | -0.01 | 0.01 | -0.66 | 0.508 |
|  | affect | 0.24 | 0.04 | 6.80 | 0 |
|  | loneliness | -0.09 | 0.04 | -2.23 | 0.026 |
|  | affect |  |  |  |  |
|  | interaction mode (ref. FtF) | -0.02 | 0.01 | -2.19 | 0.029 |
| loneliness |  |  |  |  |  |
|  | interaction mode (ref. FtF) | 0.05 | 0.01 | 3.69 | 0 |
| affect |  |  |  |  |  |
|  | loneliness | -0.16 | 0.04 | -4.00 | 0 |
| *Intercepts* |  |  |  |  |  |
|  |  | Estimate | *SE* | *z* | *p* |
|  | quality item 1 | 5.00 | 0.05 | 97.65 | 0 |
|  | quality item 3 | 4.80 | 0.05 | 103.09 | 0 |
|  | affect | 4.77 | 0.05 | 105.63 | 0 |
|  | loneliness | 2.73 | 0.06 | 47.75 | 0 |
|  | interaction mode (ref. FtF) | 0.36 | 0.02 | 24.25 | 0 |
| *Variances* |  |  |  |  |  |
|  |  | Estimate | *SE* | *z* | *p* |
|  | quality item 1 | 0.50 | 0.06 | 9.12 | 0 |
|  | quality item 2 | 0.55 | 0.06 | 9.42 | 0 |
|  | affect | 0.38 | 0.04 | 8.96 | 0 |
|  | loneliness | 0.61 | 0.07 | 9.00 | 0 |
|  | mode | 0.04 | 0.01 | 8.24 | 0 |
| *Mediation parameters* | |  |  |  |  |
|  |  | Estimate | *SE* | *z* | *p* |
|  | indirect effect affect (within person) | 0.00 | 0.01 | 0.20 | 0.842 |
|  | indirect effect loneliness (within person) | 0.00 | 0.01 | -0.20 | 0.842 |
|  | total effect affect (within person) | -0.31 | 0.04 | -8.46 | 0 |
|  | total effect loneliness (within person) | 0.69 | 0.04 | 16.00 | 0 |

**Table S3**

*Detailed Multilevel SEM Results of Sample II*

| **Level 1 (within-person)** | |  |  |  |  |
| --- | --- | --- | --- | --- | --- |
| *Latent Variables* | |  |  |  |  |
| Latent factor | Manifest variables | Estimate | *SE* | *z* | *p* |
| interaction quality latent | |  |  |  |  |
|  | quality item 1 | 1.00 |  |  |  |
|  | quality item 2 | 0.84 | 0.02 | 38.44 | 0 |
|  | quality item 3 | 0.93 | 0.04 | 25.19 | 0 |
|  | quality item 4 | 0.58 | 0.03 | 21.40 | 0 |
| positive affect latent | |  |  |  |  |
|  | positive affect item 1 | 1.00 |  |  |  |
|  | positive affect item 2 | 0.77 | 0.05 | 15.25 | 0 |
|  | positive affect item 3 | 1.14 | 0.06 | 19.58 | 0 |
| negative affect latent | |  |  |  |  |
|  | negative affect item 1 | 1.00 |  |  |  |
|  | negative affect item 2 | 1.14 | 0.08 | 13.94 | 0 |
|  | negative affect item 3 | 1.53 | 0.11 | 13.67 | 0 |
|  | negative affect item 4 | 0.98 | 0.07 | 13.90 | 0 |
| *Regressions* |  |  |  |  |  |
| Dependent variable | Independent variable | Estimate | *SE* | *z* | *p* |
| positive affect latent | |  |  |  |  |
|  | interaction mode (ref. FtF) | -0.15 | 0.10 | -1.56 | 0.119 |
|  | interaction quality latent | 0.50 | 0.03 | 16.01 | 0 |
| negative affect latent | |  |  |  |  |
|  | interaction mode (ref. FtF) | 0.15 | 0.09 | 1.60 | 0.110 |
|  | interaction quality latent | -0.26 | 0.03 | -9.45 | 0 |
| interaction quality latent | |  |  |  |  |
|  | interaction mode (ref. FtF) | 0.27 | 0.11 | 2.44 | 0.015 |
| *Covariances* | |  |  |  |  |
| Dependent variable | Independent variable | Estimate | *SE* | *z* | *p* |
| positive affect latent | |  |  |  |  |
|  | negative affect latent | -0.96 | 0.08 | -11.99 | 0 |
| negative affect item 2 | |  |  |  |  |
|  | negative affect item 3 | 1.05 | 0.15 | 7.19 | 0 |
| positive affect item 1 | |  |  |  |  |
|  | negative affect item 1 | -0.54 | 0.07 | -7.45 | 0 |
|  | positive affect item 3 | 0.74 | 0.09 | 8.37 | 0 |
| *Intercepts* |  |  |  |  |  |
|  |  | Estimate |  |  |  |
|  | quality item 1 | 0.00 |  |  |  |
|  | quality item 2 | 0.00 |  |  |  |
|  | quality item 3 | 0.00 |  |  |  |
|  | quality item 4 | 0.00 |  |  |  |
|  | positive affect item 1 | 0.00 |  |  |  |
|  | positive affect item 2 | 0.00 |  |  |  |
|  | positive affect item 3 | 0.00 |  |  |  |
|  | negative affect item 1 | 0.00 |  |  |  |
|  | negative affect item 2 | 0.00 |  |  |  |
|  | negative affect item 3 | 0.00 |  |  |  |
|  | negative affect item 4 | 0.00 |  |  |  |
|  | interaction quality latent | 0.00 |  |  |  |
|  | positive affect latent | 0.00 |  |  |  |
|  | negative affect latent | 0.00 |  |  |  |
| *Variances* |  |  |  |  |  |
|  |  | Estimate | *SE* | *z* | *p* |
|  | quality item 1 | 0.47 | 0.04 | 10.88 | 0 |
|  | quality item 2 | 0.57 | 0.04 | 16.06 | 0 |
|  | quality item 3 | 2.92 | 0.12 | 23.94 | 0 |
|  | quality item 4 | 1.70 | 0.07 | 24.68 | 0 |
|  | positive affect item 1 | 1.34 | 0.08 | 16.19 | 0 |
|  | positive affect item 2 | 4.57 | 0.19 | 24.02 | 0 |
|  | positive affect item 3 | 2.43 | 0.13 | 18.20 | 0 |
|  | negative affect item 1 | 2.67 | 0.13 | 21.22 | 0 |
|  | negative affect item 2 | 2.68 | 0.14 | 18.83 | 0 |
|  | negative affect item 3 | 4.04 | 0.23 | 17.87 | 0 |
|  | negative affect item 4 | 2.67 | 0.12 | 21.86 | 0 |
|  | interaction quality latent | 2.21 | 0.11 | 20.32 | 0 |
|  | positive affect latent | 1.12 | 0.09 | 12.21 | 0 |
|  | negative affect latent | 0.99 | 0.11 | 8.85 | 0 |
| *R-squared* |  |  |  |  |  |
|  |  | Estimate |  |  |  |
|  | quality item 1 | 0.82 |  |  |  |
|  | quality item 2 | 0.74 |  |  |  |
|  | quality item 3 | 0.40 |  |  |  |
|  | quality item 4 | 0.31 |  |  |  |
|  | positive affect item 1 | 0.55 |  |  |  |
|  | positive affect item 2 | 0.18 |  |  |  |
|  | positive affect item 3 | 0.47 |  |  |  |
|  | negative affect item 1 | 0.30 |  |  |  |
|  | negative affect item 2 | 0.36 |  |  |  |
|  | negative affect item 3 | 0.40 |  |  |  |
|  | negative affect item 4 | 0.29 |  |  |  |
|  | interaction quality latent | 0.01 |  |  |  |
|  | positive affect latent | 0.33 |  |  |  |
|  | negative affect latent | 0.14 |  |  |  |
| **Level 2 (between-person)** | |  |  |  |  |
| *Covariances* | |  |  |  |  |
| Dependent variable | Independent variable | Estimate | *SE* | *z* | *p* |
| quality item 1 | |  |  |  |  |
|  | quality item 2 | 0.77 | 0.25 | 3.07 | 0.002 |
|  | quality item 3 | 0.65 | 0.28 | 2.29 | 0.022 |
|  | quality item 4 | 0.63 | 0.24 | 2.63 | 0.009 |
|  | positive affect item 1 | 0.66 | 0.28 | 2.40 | 0.016 |
|  | positive affect item 2 | 0.19 | 0.24 | 0.82 | 0.414 |
|  | positive affect item 3 | 0.53 | 0.34 | 1.57 | 0.116 |
|  | negative affect item 1 | -0.39 | 0.29 | -1.36 | 0.175 |
|  | negative affect item 2 | -0.47 | 0.36 | -1.30 | 0.194 |
|  | negative affect item 3 | -0.41 | 0.31 | -1.30 | 0.192 |
|  | negative affect item 4 | -0.45 | 0.30 | -1.52 | 0.127 |
|  | interaction mode (ref. FtF) | -0.03 | 0.04 | -0.65 | 0.514 |
| quality item 2 | |  |  |  |  |
|  | quality item 3 | 0.75 | 0.30 | 2.47 | 0.013 |
|  | quality item 4 | 0.72 | 0.25 | 2.82 | 0.005 |
|  | positive affect item 1 | 0.60 | 0.28 | 2.18 | 0.029 |
|  | positive affect item 2 | 0.12 | 0.24 | 0.48 | 0.632 |
|  | positive affect item 3 | 0.48 | 0.35 | 1.38 | 0.167 |
|  | negative affect item 1 | -0.28 | 0.29 | -0.97 | 0.332 |
|  | negative affect item 2 | -0.39 | 0.37 | -1.05 | 0.295 |
|  | negative affect item 3 | -0.29 | 0.32 | -0.91 | 0.363 |
|  | negative affect item 4 | -0.36 | 0.30 | -1.20 | 0.232 |
|  | interaction mode (ref. FtF) | -0.02 | 0.04 | -0.52 | 0.601 |
| quality item 3 | |  |  |  |  |
|  | quality item 4 | 0.76 | 0.32 | 2.39 | 0.017 |
|  | positive affect item 1 | 0.10 | 0.33 | 0.31 | 0.76 |
|  | positive affect item 2 | -0.08 | 0.32 | -0.23 | 0.816 |
|  | positive affect item 3 | 0.06 | 0.44 | 0.13 | 0.9 |
|  | negative affect item 1 | 0.36 | 0.39 | 0.93 | 0.354 |
|  | negative affect item 2 | 0.15 | 0.48 | 0.31 | 0.758 |
|  | negative affect item 3 | 0.03 | 0.42 | 0.07 | 0.944 |
|  | negative affect item 4 | 0.11 | 0.39 | 0.27 | 0.787 |
|  | interaction mode (ref. FtF) | 0.10 | 0.06 | 1.65 | 0.098 |
| quality item 4 | |  |  |  |  |
|  | positive affect item 1 | 0.45 | 0.28 | 1.64 | 0.1 |
|  | positive affect item 2 | -0.21 | 0.26 | -0.80 | 0.422 |
|  | positive affect item 3 | 0.54 | 0.37 | 1.45 | 0.146 |
|  | negative affect item 1 | -0.30 | 0.31 | -0.96 | 0.335 |
|  | negative affect item 2 | -0.58 | 0.40 | -1.44 | 0.151 |
|  | negative affect item 3 | -0.27 | 0.34 | -0.81 | 0.418 |
|  | negative affect item 4 | -0.50 | 0.33 | -1.53 | 0.125 |
|  | interaction mode (ref. FtF) | 0.02 | 0.04 | 0.39 | 0.7 |
| positive affect item 1 | |  |  |  |  |
|  | positive affect item 2 | 0.52 | 0.33 | 1.57 | 0.116 |
|  | positive affect item 3 | 1.50 | 0.54 | 2.80 | 0.005 |
|  | negative affect item 1 | -1.31 | 0.47 | -2.80 | 0.005 |
|  | negative affect item 2 | -1.66 | 0.59 | -2.83 | 0.005 |
|  | negative affect item 3 | -1.14 | 0.47 | -2.42 | 0.015 |
|  | negative affect item 4 | -1.06 | 0.44 | -2.41 | 0.016 |
|  | interaction mode (ref. FtF) | -0.13 | 0.06 | -2.16 | 0.031 |
| positive affect item 2 | |  |  |  |  |
|  | positive affect item 3 | 0.49 | 0.44 | 1.13 | 0.258 |
|  | negative affect item 1 | -0.12 | 0.37 | -0.32 | 0.748 |
|  | negative affect item 2 | 0.00 | 0.46 | 0.01 | 0.996 |
|  | negative affect item 3 | 0.03 | 0.39 | 0.07 | 0.945 |
|  | negative affect item 4 | 0.24 | 0.37 | 0.65 | 0.513 |
|  | interaction mode (ref. FtF) | -0.06 | 0.05 | -1.11 | 0.269 |
| positive affect item 3 | |  |  |  |  |
|  | negative affect item 1 | -1.24 | 0.57 | -2.17 | 0.03 |
|  | negative affect item 2 | -2.18 | 0.79 | -2.76 | 0.006 |
|  | negative affect item 3 | -1.68 | 0.66 | -2.56 | 0.011 |
|  | negative affect item 4 | -1.24 | 0.58 | -2.14 | 0.032 |
|  | interaction mode (ref. FtF) | -0.11 | 0.08 | -1.52 | 0.128 |
| negative affect item 1 | |  |  |  |  |
|  | negative affect item 2 | 2.10 | 0.71 | 2.95 | 0.003 |
|  | negative affect item 3 | 1.35 | 0.56 | 2.41 | 0.016 |
|  | negative affect item 4 | 1.64 | 0.57 | 2.89 | 0.004 |
|  | interaction mode (ref. FtF) | 0.17 | 0.07 | 2.34 | 0.019 |
| negative affect item 2 | |  |  |  |  |
|  | negative affect item 3 | 2.00 | 0.74 | 2.71 | 0.007 |
|  | negative affect item 4 | 1.96 | 0.70 | 2.80 | 0.005 |
|  | interaction mode (ref. FtF) | 0.11 | 0.08 | 1.39 | 0.166 |
| negative affect item 3 | |  |  |  |  |
|  | negative affect item 4 | 1.36 | 0.56 | 2.40 | 0.016 |
|  | interaction mode (ref. FtF) | 0.06 | 0.07 | 0.90 | 0.37 |
| negative affect item 4 | |  |  |  |  |
|  | interaction mode (ref. FtF) | 0.09 | 0.07 | 1.32 | 0.188 |
| *Intercepts* |  |  |  |  |  |
|  |  | Estimate | *SE* | *z* | *p* |
|  | quality item 1 | 8.13 | 0.19 | 42.22 | 0 |
|  | quality item 2 | 8.19 | 0.20 | 41.12 | 0 |
|  | quality item 3 | 7.10 | 0.27 | 26.40 | 0 |
|  | quality item 4 | 8.37 | 0.21 | 39.33 | 0 |
|  | positive affect item 1 | 7.26 | 0.26 | 28.29 | 0 |
|  | positive affect item 2 | 5.86 | 0.26 | 23.00 | 0 |
|  | positive affect item 3 | 6.59 | 0.35 | 18.72 | 0 |
|  | negative affect item 1 | 1.98 | 0.30 | 6.50 | 0 |
|  | negative affect item 2 | 2.32 | 0.38 | 6.07 | 0 |
|  | negative affect item 3 | 3.05 | 0.33 | 9.20 | 0 |
|  | negative affect item 4 | 1.51 | 0.31 | 4.85 | 0 |
|  | interaction mode (ref. FtF) | 0.23 | 0.04 | 5.48 | 0 |
| *Variances* |  |  |  |  |  |
|  |  | Estimate | *SE* | *z* | *p* |
|  | quality item 1 | 0.76 | 0.25 | 3.08 | 0.003 |
|  | quality item 2 | 0.83 | 0.26 | 3.14 | 0.002 |
|  | quality item 3 | 1.49 | 0.48 | 3.11 | 0.002 |
|  | quality item 4 | 0.95 | 0.30 | 3.14 | 0.002 |
|  | positive affect item 1 | 1.39 | 0.44 | 3.16 | 0.002 |
|  | positive affect item 2 | 1.32 | 0.43 | 3.05 | 0.003 |
|  | positive affect item 3 | 2.64 | 0.82 | 3.21 | 0.002 |
|  | negative affect item 1 | 1.96 | 0.62 | 3.18 | 0.002 |
|  | negative affect item 2 | 3.14 | 0.97 | 3.23 | 0.002 |
|  | negative affect item 3 | 2.27 | 0.72 | 3.14 | 0.002 |
|  | negative affect item 4 | 2.04 | 0.64 | 3.20 | 0.002 |
|  | interaction mode (ref. FtF) | 0.04 | 0.01 | 3.00 | 0.003 |
| *Mediation parameters* | |  |  |  |  |
|  |  | Estimate | *SE* | *z* | *p* |
|  | indirect effect positive affect (within person) | 0.13 | 0.06 | 2.41 | 0.016 |
|  | indirect effect negative affect (within person) | -0.07 | 0.03 | -2.36 | 0.018 |
|  | total effect positive affect (within person) | -0.02 | 0.11 | -0.18 | 0.860 |
|  | total effect negative affect (within person) | 0.07 | 0.09 | 0.79 | 0.429 |

**Table S2**

*Fixed Effects Within-Person Multilevel SEM Results of Sample I With Manifest Variables and Between-Person Effects*

| Dependent variable | Independent variable | Estimate | SE | z | p |
| --- | --- | --- | --- | --- | --- |
| *Within-person effects* |  |  |  |  |  |
| Interaction quality |  |  |  |  |  |
|  | Interaction mode (ref. FtF) | -0.005 | 0.030 | -0.168 | .866 |
| Affect valence |  |  |  |  |  |
|  | Interaction mode (ref. FtF) | -0.308 | 0.035 | -8.845 | < .001 |
|  | Interaction quality | 0.380 | 0.017 | 22.545 | < .001 |
| Loneliness |  |  |  |  |  |
|  | Interaction mode (ref. FtF) | 0.686 | 0.042 | 16.49 | < .001 |
|  | Interaction quality | -0.365 | 0.020 | -18.131 | < .001 |
| *Between-person effects* | |  |  |  |  |
| Interaction quality |  |  |  |  |  |
|  | Interaction mode (ref. FtF) | -0.426 | 0.274 | -1.556 | .120 |
|  | Gender (ref. male) | 0.088 | 0.129 | 0.684 | .494 |
| Affect valence |  |  |  |  |  |
|  | Interaction mode (ref. FtF) | -0.318 | 0.207 | -1.539 | .124 |
|  | Interaction quality | 0.596 | 0.056 | 10.653 | < .001 |
|  | Gender (ref. male) | -0.054 | 0.096 | -0.560 | .575 |
| Loneliness |  |  |  |  |  |
|  | Interaction mode (ref. FtF) | 1.154 | 0.310 | 3.723 | < .001 |
|  | Interaction quality | -0.294 | 0.084 | -3.509 | < .001 |
|  | Gender (ref. male) | -0.016 | 0.144 | -0.110 | .913 |

*Note*. *N* = 216, *N_obs_* = 5116. More detailed model results can be found in the R-Markdown file on  [https://osf.io/dezuw/](https://osf.io/dezuw/?view_only=f142e478eb324771beef65e91b716f90).

**(Preregistered) Robustness Analyses**

We conducted a series of (preregistered) robustness analyses. First, we re-estimated our multilevel mediation SEMs with manifest variables (instead of latent ones), which allowed us to jointly estimate between-person coefficients. For detailed results of these multilevel SEMs, see Supplementary Materials Table S4-S5. The effect sizes and significance levels of the within-person effects are similar to the ones reported above. The negative effect of interaction mode on interaction quality in Sample II increased in size and significance (*b* = 0.39, *SE* = 0.10, *p* < .001). On the between-person level, the model estimated a positive association between interaction mode and loneliness (*b* = 1.15, *SE* = 0.31, *p* < .001) and interaction quality and affect (*b* = 0.60, *SE* = 0.06, *p* < .001), as well as a negative association between interaction quality and loneliness (*b* = -.29, *SE* = 0.08, *p* < .001).

Second, we estimated multilevel regression models [(Snijders & Bosker, 1999)](https://www.zotero.org/google-docs/?ZtYDqu), in which we could include random slope terms for the focal within-person effects, which is not possible to estimate in the current version of lavaan [(Rosseel, 2012)](https://www.zotero.org/google-docs/?gqbUML). Effect sizes and significance levels are similar to the results reported above, besides that the within-person effect of interaction mode on interaction quality in Sample II is non-significant (*b* = 0.27, *SE* = 0.31, *p* = .391). Detailed model results are reported in Supplementary Materials Table S6 and S7.

Third, we re-estimated the Sample I model by additionally controlling for the effect of previous momentary well-being (i.e., lag-1) on current well-being and interaction quality. Consequently, we are modeling *change* in momentary well-being [(Castro-Schilo & Grimm, 2018)](https://www.zotero.org/google-docs/?PNZyrz). The within-person fixed effects of this SEM is presented in Supplementary Materials Table S8. The results are similar in size and significance level to the ones presented in the main analyses.

Fourth, we re-estimated the main model of Sample I with only the observations of modes FtF, Calls, and Videocalls to match the provided categories in Sample II. This way, we can examine whether the differences in results between the two samples are a consequence of different answer categories provided. Detailed model results of this robustness analysis are reported in Supplementary Materials Table S9. Also in this robustness analysis, the effect sizes are similar in size and significance level to the ones presented above.

Fifth, in Sample II, we additionally estimated a model in which we control for interaction partner closeness and the number of interaction partners, as this might affect the quality of social interactions and subsequent well-being. The results of this robustness analysis are reported in Supplementary Materials Table S10. The main results were not affected by the inclusion of these variables in the model. Interaction-partner closeness was, on a within-person level, associated with higher interaction quality (*b* = 0.35, *SE* = 0.06, *p* < .001) and lower levels of positive affect (*b* = -0.12, *SE* = 0.06, *p* = .041).

**Table S3**

*Fixed Effects Within-Person Multilevel SEM Results of Sample II With Manifest Variables and Between-Person Effects*

| Dependent variable | Independent variable | Estimate | *SE* | *z* | *p* |
| --- | --- | --- | --- | --- | --- |
| *Within-person effects* |  |  |  |  |  |
| Interaction quality |  |  |  |  |  |
|  | Interaction mode (ref. FtF) | 0.40 | 0.10 | 4.07 | < .001 |
| Positive affect |  |  |  |  |  |
|  | Interaction mode (ref. FtF) | -0.13 | 0.11 | -1.26 | .207 |
|  | Interaction quality | 0.45 | 0.03 | 15.33 | < .001 |
| Negative affect |  |  |  |  |  |
|  | Interaction mode (ref. FtF) | 0.21 | 0.11 | 1.98 | .048 |
|  | Interaction quality | -0.28 | 0.03 | -9.62 | < .001 |
| *Between-person effects* | |  |  |  |  |
| Interaction quality |  |  |  |  |  |
|  | Interaction mode (ref. FtF) | 0.40 | 1.07 | 0.37 | .709 |
|  | Gender (ref. male) | -0.02 | 0.46 | -0.03 | .974 |
| Positive affect |  |  |  |  |  |
|  | Interaction mode (ref. FtF) | -2.87 | 1.07 | -2.69 | .007 |
|  | Interaction quality | 0.42 | 0.22 | 1.90 | .057 |
|  | Gender (ref. male) | -0.09 | 0.46 | -0.19 | .849 |
| Negative affect |  |  |  |  |  |
|  | Interaction mode (ref. FtF) | 2.93 | 1.47 | 2.00 | .046 |
|  | Interaction quality | -0.38 | 0.31 | -1.25 | .211 |
|  | Gender (ref. male) | -0.20 | 0.64 | -0.31 | .756 |

*Note*. *N* = 21, *N_obs_* = 1147. More detailed model results can be found in the R-Markdown file on https://osf.io/dezuw/.

**Table S3**

*Multilevel Models of Sample I*

|  |  | **Interaction Quality** | | |  | **Affect Valence** | | |  | **Loneliness** | | |
| --- | --- | --- | --- | --- | --- | --- | --- | --- | --- | --- | --- | --- |
| *Predictors* |  | *b* | *CI* | *p* |  | *b* | *CI* | *p* |  | *b* | *CI* | *p* |
| Intercept |  | 5.04 | 4.76 – 5.31 | **<.001** |  | 2.06 | 1.52 – 2.61 | **<.001** |  | 3.97 | 3.16 – 4.77 | **<.001** |
| Interaction mode between-person (ref. FtF) | | -0.38 | -0.80 – 0.05 | .082 |  | -0.29 | -0.62 – 0.03 | .075 |  | 0.98 | 0.50 – 1.45 | **<.001** |
| Interaction mode within-person (ref. FtF) | | -0.01 | -0.09 – 0.07 | .84 |  | -0.31 | -0.39 – -0.23 | **<.001** |  | 0.7 | 0.59 – 0.81 | **<.001** |
| Gender (ref. male) |  | 0.09 | -0.17 – 0.34 | .507 |  | -0.04 | -0.23 – 0.15 | .673 |  | -0.01 | -0.29 – 0.27 | .939 |
| Interaction quality between-person |  |  |  |  |  | 0.57 | 0.47 – 0.67 | **<.001** |  | -0.32 | -0.47 – -0.17 | **<.001** |
| Interaction quality within-person |  |  |  |  |  | 0.38 | 0.34 – 0.43 | **<.001** |  | -0.35 | -0.41 – -0.29 | **<.001** |
| **Random Effects** |  |  |  |  |  |  |  |  |  |  |  |  |
| σ^2^ |  | 0.80 |  |  |  | 1.09 |  |  |  | 1.49 |  |  |
| τ_00_ |  | 0.45 |  |  |  | 0.22 |  |  |  | 0.53 |  |  |
| τ_11_ |  | 0.14 | |  |  | 0.11 | |  |  | 0.27 | |  |
|  |  |  |  |  |  | 0.04 | |  |  | 0.10 | |  |
| ρ_01_ |  | -.11 |  |  |  | .07 |  |  |  | .28 |  |  |
|  |  |  |  |  |  | .18 |  |  |  | -.09 |  |  |
| ICC |  | .37 |  |  |  | .20 |  |  |  | .31 |  |  |
| Marginal R^2^ / Conditional R^2^ |  | .006 / .375 | |  |  | .178 / .342 | |  |  | .116 / .388 | |  |

Note. N = 216; N_obs_ = 5116; b = unstandardized coefficient, CI = 95% Confidence Interval, σ2 = level 1 residual variance, τ00 = random intercept variance, τ11 = random slope variance, ρ01 = correlation between random intercept and random slope, ICC = Intraclass correlation.

**Table S4**

*Multilevel Models of Sample II*

|  |  | **Interaction Quality** | | |  | **Positive Affect** | | |  | **Negative Affect** | | |
| --- | --- | --- | --- | --- | --- | --- | --- | --- | --- | --- | --- | --- |
| *Predictors* |  | *b* | *CI* | *p* |  | *b* | *CI* | *p* |  | *b* | *CI* | *p* |
| Intercept |  | 7.76 | 6.77 – 8.76 | **<0.001** |  | 3.95 | 0.28 – 7.62 | **0.035** |  | 4.8 | -0.18 – 9.78 | 0.059 |
| Interaction mode between-person (ref. FtF) | | 0.58 | -1.40 – 2.57 | 0.565 |  | -2.65 | -4.68 – -0.61 | **0.011** |  | 2.74 | -0.03 – 5.52 | 0.053 |
| Interaction mode within-person (ref. FtF) | | 0.29 | -0.29 – 0.88 | 0.326 |  | -0.09 | -0.44 – 0.26 | 0.608 |  | 0.17 | -0.11 – 0.46 | 0.233 |
| Gender (ref. male) |  | 0.05 | -0.91 – 1.01 | 0.918 |  | -0.02 | -0.99 – 0.96 | 0.973 |  | -0.15 | -1.47 – 1.17 | 0.824 |
| Interaction quality between-person |  |  |  |  |  | 0.41 | -0.04 – 0.86 | 0.072 |  | -0.39 | -1.00 – 0.22 | 0.206 |
| Interaction quality within-person |  |  |  |  |  | 0.43 | 0.33 – 0.53 | **<0.001** |  | -0.28 | -0.35 – -0.21 | **<0.001** |
| **Random Effects** |  |  |  |  |  |  |  |  |  |  |  |  |
| σ^2^ |  | 1.80 |  |  |  | 2.15 |  |  |  | 2.22 |  |  |
| τ_00_ |  | 0.91 |  |  |  | 0.93 |  |  |  | 1.82 |  |  |
| τ_11_ |  | 1.59 | |  |  | 0.36 | |  |  | 0.16 | |  |
|  |  |  |  |  |  | 0.03 | |  |  | 0.01 | |  |
| ρ_01_ |  | -.24 |  |  |  | -.13 |  |  |  | -.28 |  |  |
|  |  |  |  |  |  | -.19 |  |  |  | -.35 |  |  |
| ICC |  | .39 |  |  |  | .33 |  |  |  | .46 |  |  |
| Marginal R^2^ / Conditional R^2^ |  | .008 / .394 | |  |  | .162 / .435 | |  |  | .097 / .508 | |  |

Note. N = 22; N_obs = 1386; b = unstandardized coefficient, CI = 95% Confidence Interval, σ2 = level 1 residual variance, τ00 = random intercept variance, τ11 = random slope variance, ρ01 = correlation between random intercept and random slope, ICC = Intraclass correlation.

**Table S5**

*Fixed Effects Within-Person Multilevel SEM Results of Sample I*

| Dependent variable | Independent variable | Estimate | *SE* | *z* | *p* |
| --- | --- | --- | --- | --- | --- |
| Interaction quality (latent) | |  |  |  |  |
|  | Interaction mode (ref. FtF) | -0.023 | 0.047 | -0.486 | .627 |
|  | Previous (lag-1) affect | 0.051 | 0.019 | 2.646 | .008 |
|  | Previous (lag-1) loneliness | -0.036 | 0.016 | -2.228 | .026 |
| Affect valence |  |  |  |  |  |
|  | Interaction mode (ref. FtF) | -0.347 | 0.045 | -7.786 | < .001 |
|  | Previous (lag-1) affect | 0.208 | 0.017 | 12.33 | < .001 |
|  | Interaction quality (latent) | 0.326 | 0.020 | 15.928 | < .001 |
| Loneliness |  |  |  |  | < .001 |
|  | Interaction mode (ref. FtF) | 0.649 | 0.052 | 12.409 | < .001 |
|  | Previous (lag-1) loneliness | 0.133 | 0.017 | 8.073 | < .001 |
|  | Interaction quality (latent) | -0.343 | 0.024 | -14.329 | < .001 |

*Note*. *N* = 215, *N_obs_* = 3249, CFI= .90, SRMR = .022, RMSEA = .089. More detailed model results can be found in the R-Markdown file on https://osf.io/dezuw/.

**Table S6**

*Fixed Effects Within-Person Multilevel SEM Results of Sample I with Reduced Mode Categories (i.e., Texting and Social Media Interactions Were Removed)*

| Dependent variable | Independent variable | Estimate | *SE* | *z* | *p* |
| --- | --- | --- | --- | --- | --- |
| Affect valence |  |  |  |  |  |
|  | Interaction mode (ref. FtF) | -0.297 | 0.056 | -5.318 | < .001 |
|  | Interaction Quality (latent) | 0.384 | 0.019 | 20.46 | < .001 |
| Loneliness |  |  |  |  | < .001 |
|  | Interaction mode (ref. FtF) | 0.577 | 0.065 | 8.85 | < .001 |
|  | Interaction Quality (latent) | -0.374 | 0.021 | -17.441 | < .001 |
| Interaction Quality (latent) | |  |  |  |  |
|  | Interaction mode (ref. FtF) | 0.087 | 0.059 | 1.465 | .143 |

*Note*. *N* = 216, *N_obs_* = 3890, CFI= .99, SRMR = .019, RMSEA = .044. More detailed model results can be found in the R-Markdown file on https://osf.io/dezuw/.

**Table S7**

*Fixed Effects Within-Person Multilevel SEM Results of Sample II with Additional Covariates*

| Dependent variable | Independent variable | Estimate | *SE* | *z* | *p* |
| --- | --- | --- | --- | --- | --- |
| Positive Affect (latent) | |  |  |  |  |
|  | Interaction mode (ref. FtF) | -0.056 | 0.112 | -0.496 | .620 |
|  | Interaction Quality (latent) | 0.549 | 0.038 | 14.624 | < .001 |
|  | Number of interaction partners | 0.008 | 0.042 | 0.192 | .848 |
|  | Interaction partner closeness | -0.116 | 0.057 | -2.04 | .041 |
| Negative Affect (latent) | |  |  |  |  |
|  | Interaction mode (ref. FtF) | 0.099 | 0.105 | 0.94 | .347 |
|  | Interaction Quality (latent) | -0.307 | 0.034 | -8.956 | < .001 |
|  | Number of interaction partners | -0.028 | 0.039 | -0.725 | .469 |
|  | Interaction partner closeness | 0.076 | 0.053 | 1.437 | .151 |
| Interaction Quality (latent) | |  |  |  |  |
|  | Interaction mode (ref. FtF) | 0.125 | 0.117 | 1.071 | .284 |
|  | Number of interaction partners | -0.050 | 0.044 | -1.145 | .252 |
|  | Interaction partner closeness | 0.351 | 0.057 | 6.124 | < .001 |

*Note*. *N* = 21, *N_obs_* = 1147, CFI= .92, SRMR = .048, RMSEA = .062. More detailed model results can be found in the R-Markdown file on https://osf.io/dezuw/. **Table S8**

*Fixed Effects Within-Person Multilevel SEM Results of Sample I with Moderation Effect*

| Dependent variable | Independent variable | Estimate | SE | z | p |
| --- | --- | --- | --- | --- | --- |
| Affect valence |  |  |  |  |  |
|  | Interaction mode (ref. FtF) | 0.138 | 0.155 | 0.892 | .373 |
|  | Interaction Quality | 0.411 | 0.02 | 20.733 | < .001 |
|  | Interaction mode * Interaction quality | -0.089 | 0.03 | -2.956 | .003 |
| Loneliness |  |  |  |  |  |
|  | Interaction mode (ref. FtF) | 0.014 | 0.185 | 0.075 | .94 |
|  | Interaction Quality | -0.411 | 0.024 | -17.39 | < .001 |
|  | Interaction mode * Interaction quality | 0.134 | 0.036 | 3.729 | < .001 |

*Note*. *N* = 216, *N_obs_* = 5116, More detailed model results can be found in the R-Markdown file on https://osf.io/dezuw/.

**Table S9**

*Fixed Effects Within-Person Multilevel SEM Results of Sample II with Moderation Effect*

| Dependent variable | Independent variable | Estimate | SE | z | p |
| --- | --- | --- | --- | --- | --- |
| Positive Affect |  |  |  |  |  |
|  | Interaction mode (ref. FtF) | -0.693 | 0.506 | -1.368 | .171 |
|  | Interaction Quality | 0.431 | 0.033 | 13.024 | <.001 |
|  | Interaction mode * Interaction quality | 0.068 | 0.06 | 1.13 | .258 |
| Negative Affect |  |  |  |  |  |
|  | Interaction mode (ref. FtF) | 0.386 | 0.508 | 0.761 | .447 |
|  | Interaction Quality | -0.277 | 0.033 | -8.335 | <.001 |
|  | Interaction mode * Interaction quality | -0.022 | 0.06 | -0.357 | .721 |

*Note*. *N* = 22, *N_obs_* = 1386, More detailed model results can be found in the R-Markdown file on https://osf.io/dezuw/.

**Table S10**

*Multilevel SEM for Sample I with Different Interaction Modes Compared to Face-To-Face Interactions*

| Focal mode | Dependent Variable | Effect | Hypothesis | Estimate |  | SE | zZ | p |
| --- | --- | --- | --- | --- | --- | --- | --- | --- |
| Videocall  (Total  N_obs_ = 3556) | Interaction quality (latent) | Video call (ref. FtF) | H1 | -0.12 |  | 0.10 | -1.28 | 0.202 |
|  | affect | Video call (ref. FtF) |  | -0.24 | ** | 0.09 | -2.69 | 0.007 |
|  | affect | Interaction quality (latent) | H2 | 0.39 | *** | 0.02 | 20.04 | 0.000 |
|  | affect | Indirect within-person effect | H4 | -0.05 |  | 0.04 | -1.27 | 0.203 |
|  | affect | Total within-person effect | H3 | -0.29 | ** | 0.10 | -3.04 | 0.002 |
|  | loneliness | Video call (ref. FtF) |  | 0.24 | * | 0.10 | 2.32 | 0.020 |
|  | loneliness | Interaction quality (latent) | H2 | -0.38 | *** | 0.02 | -17.21 | 0.000 |
|  | loneliness | Indirect within-person effect | H4 | 0.05 |  | 0.04 | 1.27 | 0.203 |
|  | loneliness | Total within-person effect | H3 | 0.29 | ** | 0.11 | 2.66 | 0.008 |
| Call (Total  N_obs_ = 3664) | Interaction quality (latent) | Call (ref. FtF) | H1 | 0.17 | * | 0.07 | 2.41 | 0.016 |
|  | affect | Call (ref. FtF) |  | -0.34 | *** | 0.07 | -5.06 | 0.000 |
|  | affect | Interaction quality (latent) | H2 | 0.38 | *** | 0.02 | 19.55 | 0.000 |
|  | affect | Indirect within-person effect | H4 | 0.06 | * | 0.03 | 2.40 | 0.016 |
|  | affect | Total within-person effect | H3 | -0.27 | *** | 0.07 | -3.88 | 0.000 |
|  | loneliness | Call (ref. FtF) |  | 0.76 | *** | 0.08 | 9.82 | 0.000 |
|  | loneliness | Interaction quality (latent) | H2 | -0.38 | *** | 0.02 | -17.20 | 0.000 |
|  | loneliness | Indirect within-person effect | H4 | -0.06 | * | 0.03 | -2.39 | 0.017 |
|  | loneliness | Total within-person effect | H3 | 0.69 | *** | 0.08 | 8.63 | 0.000 |
| Texting (Total  N_obs_ = 4366) | Interaction quality (latent) | Texting (ref. FtF) | H1 | -0.01 |  | 0.04 | -0.13 | 0.894 |
|  | affect | Texting (ref. FtF) |  | -0.34 | *** | 0.04 | -8.04 | 0.000 |
|  | affect | Interaction quality (latent) | H2 | 0.36 | *** | 0.02 | 20.12 | 0.000 |
|  | affect | Indirect within-person effect | H4 | 0.00 |  | 0.02 | -0.13 | 0.894 |
|  | affect | Total within-person effect | H3 | -0.34 | *** | 0.04 | -7.71 | 0.000 |
|  | loneliness | Texting (ref. FtF) |  | 0.78 | *** | 0.05 | 15.64 | 0.000 |
|  | loneliness | Interaction quality (latent) | H2 | -0.36 | *** | 0.02 | -17.31 | 0.000 |
|  | loneliness | Indirect within-person effect | H4 | 0.00 |  | 0.02 | 0.13 | 0.894 |
|  | loneliness | Total within-person effect | H3 | 0.78 | *** | 0.05 | 15.13 | 0.000 |
| Social media (Total  N_obs_ = 3520) | Interaction quality (latent) | Social Media (ref. FtF) | H1 | -0.17 |  | 0.09 | -1.75 | 0.081 |
|  | affect | Social Media (ref. FtF) |  | -0.21 | * | 0.09 | -2.36 | 0.018 |
|  | affect | Interaction quality (latent) | H2 | 0.37 | *** | 0.02 | 18.91 | 0.000 |
|  | affect | Indirect within-person effect | H4 | -0.06 |  | 0.04 | -1.74 | 0.082 |
|  | affect | Total within-person effect | H3 | -0.27 | ** | 0.09 | -2.89 | 0.004 |
|  | loneliness | Social Media (ref. FtF) |  | 0.63 | *** | 0.10 | 6.05 | 0.000 |
|  | loneliness | Interaction quality (latent) | H2 | -0.36 | *** | 0.02 | -15.87 | 0.000 |
|  | loneliness | Indirect within-person effect | H4 | 0.06 |  | 0.03 | 1.74 | 0.082 |
|  | loneliness | Total within-person effect | H3 | 0.69 | *** | 0.11 | 6.38 | 0.000 |

**Table S11**

*Overview of the used measures in the two samples*

| Measure | Sample I | Sample II |
| --- | --- | --- |
| Interaction mode | - How have you conducted the most recent social interaction that happened within the last 10 minutes?  1 = face-to-face  2 = “phone call”, “video call”, “social media”, or “text or chat” | - Which type of interaction did you have?  1 = face-to-face  2 = “phone call” or “video call” |
| Interaction quality | - Did you feel valued by your interaction partner in your most recent social interaction?  - Do you think your interaction partner felt valued by you in your most recent social interaction?  - Do you consider that after your most recent social interaction, the relationship with your interaction partner… worsened/strengthened? | - I enjoyed the interaction.  - I felt like my interaction partner(s) enjoyed the interaction  - This interaction was meaningful for me  - During this interaction I could be myself |
| Momentary well-being | - Affect valence: How do you feel right now?  - Loneliness: How lonely do you feel at the moment? | - Positive affect: “I feel happy”, “I feel energetic”, and “I feel relaxed”.  - Negative affect: “I feel sad”, “I feel anxious”, “I feel stressed”, and “I feel irritated” |
| Number of interaction partners |  | - Number of interaction partners |
| Interaction partner closeness |  | - How close are you with this person? |

**Interaction Quality and Momentary Well-Being by Specific Interaction Modes**

We further explored which interaction modes specifically were driving the moderation effect in Sample I. For this, we estimated multilevel models, predicting momentary well-being outcomes by interaction quality for each interaction mode separately. Figure S1 shows the estimated within- and between-person coefficients of such models. Worth discussing is the small and non-significant within-person effect of social media interactions with loneliness. In moments where participants would rate social media interactions of higher quality than usual, no higher affect (*b* = 0.21, *SE* = 0.11, *p* = .059) or lower loneliness (*b* = 0.33, *SE* = 0.18, *p* = .103) was reported subsequently. This null-finding might have to do with the broad spectrum of behaviors that can be reported as “social media” interactions [(Hall, 2018)](https://www.zotero.org/google-docs/?51bfUq). Also, there was no within-person association of video call quality with loneliness (*b* = -0.17, *SE* = 0.20, *p* = .421), which may be attributed to the different contexts and purposes for which video calls are used. More work on conceptual clarification in digital communication research is needed [(Birngmann et al., 2022; Elmer et al., 2025)](https://www.zotero.org/google-docs/?iFqZGV).

**Figure S1**

*Multilevel coefficients and 95% Confidence Intervals for the Interaction Quality to Momentary Well-Being Effect in Sample I (a) and Sample II (b), Differentiated by Within- and Between-Person Effect and Interaction Mode*


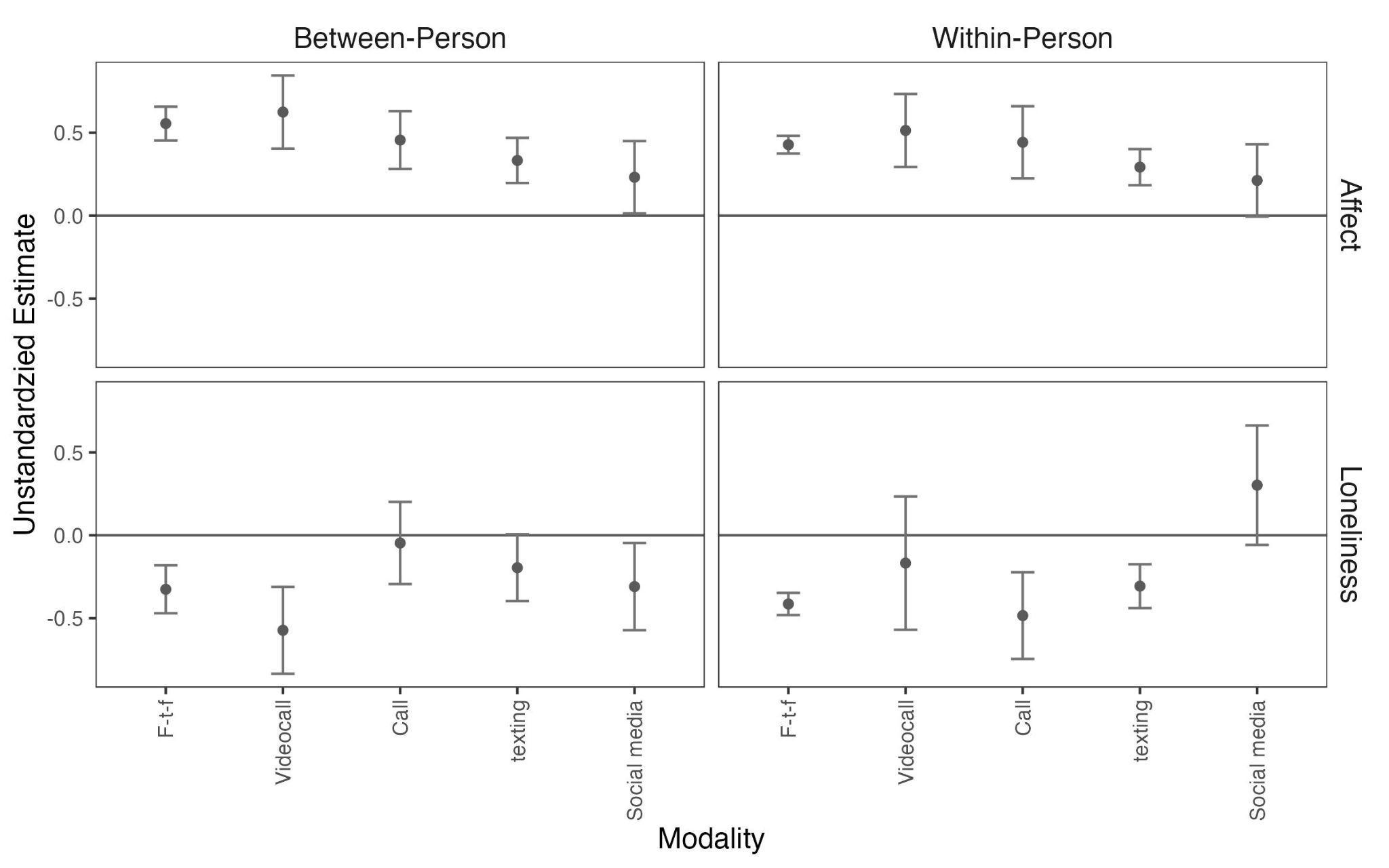


1. Sample I


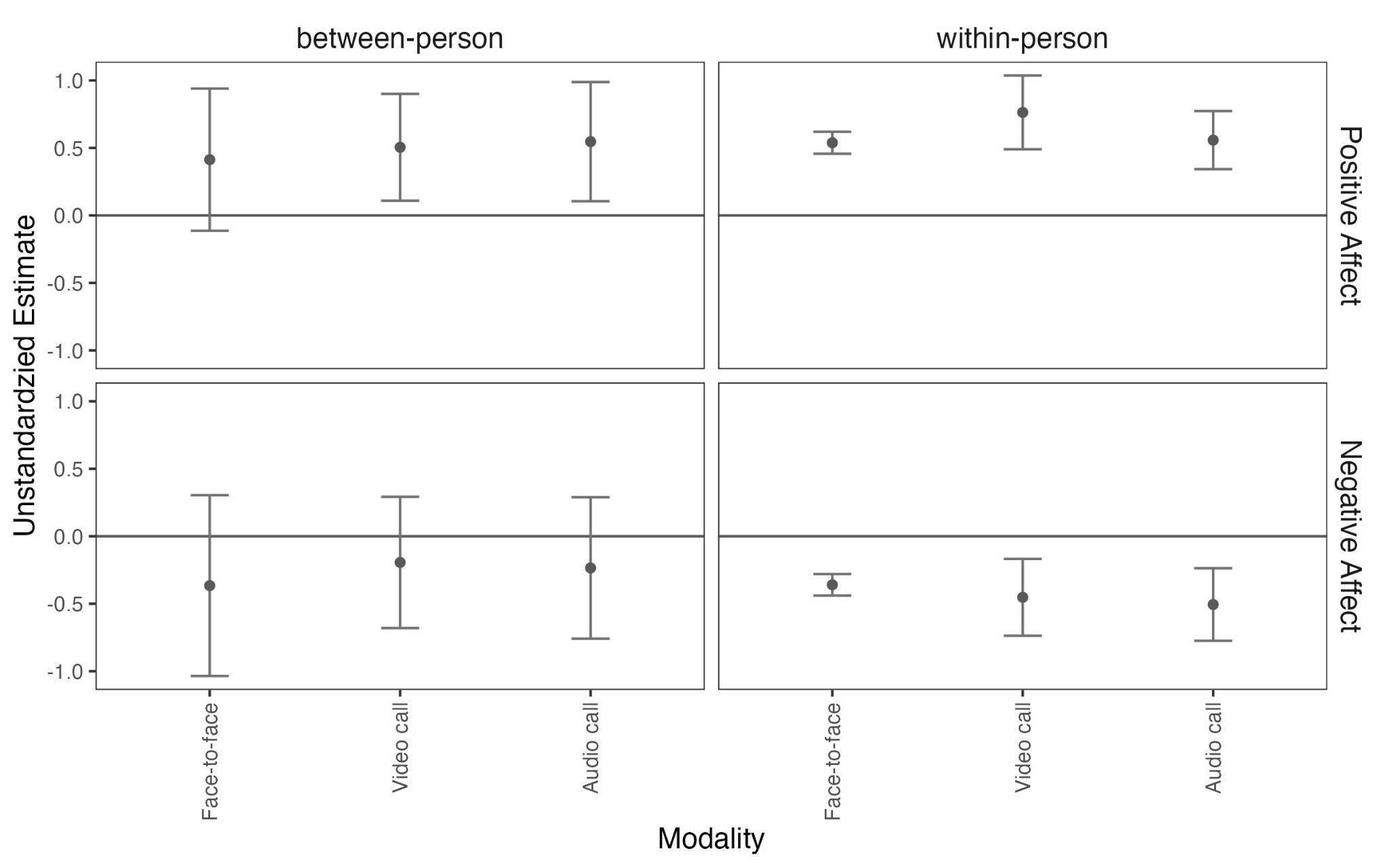


1. Sample II

**Background Information on Datasets**

This paper makes use of two data collections that pursue different goals. Data of the Spanish Sample was collected mainly to examine the effects of an intervention to reduce phone use during social interactions (https://osf.io/dezuw/). Some of the variables used in this paper were also used as part of two other manuscripts (both referenced in the article) with different purposes and involving multiple datasets in their analyses, specifically:

- Interaction mode and interaction quality was used in an analysis to examine the moderating effects of interaction partner type, interaction location, and interaction purpose [(Fernández, Elmer, et al., 2025)](https://www.zotero.org/google-docs/?akn1wS). For details, see https://osf.io/dezuw/. Momentary well-being was not used in this paper.
- Interaction quality was used as part of another multi-study paper, specifically exploring individual differences in momentary closeness and loneliness and the moderating role of the trait need to belong [(Fernández et al., 2025)](https://www.zotero.org/google-docs/?AT62Qi). Interaction mode was not part of these analyses.

Data of the Dutch Sample were collected mainly to examine the feasibility of combining Experience Sampling Methodology (ESM), personal social network data collection and passive smartphone sensing data. Interaction mode or interaction quality variables were not used in other papers, only momentary well-being variables were used as part of an analysis examining the bidirectional associations with smartphone use and context variables [(Elmer et al., 2025; Langener et al., 2024; Stadel et al., 2023)](https://www.zotero.org/google-docs/?5S6wPF).

**References**

[Birngmann, L., Elmer, T., & Eronen, M. (2022). Back to basics: The importance of conceptual clarity in psychological science. *Current Directions in Psychological Science*.](https://www.zotero.org/google-docs/?zRyqN1)

[Castro-Schilo, L., & Grimm, K. J. (2018). Using residualized change versus difference scores for longitudinal research. *Journal of Social and Personal Relationships*, *35*(1), 32–58. https://doi.org/10.1177/0265407517718387](https://www.zotero.org/google-docs/?zRyqN1)

[Elmer, T., Fernández, A., Stadel, M., Kas, M. J. H., & Langener, A. M. (2025). Bidirectional associations between smartphone usage and momentary well-being in young adults: Tackling methodological challenges by combining experience sampling methods with passive smartphone data. *Emotion*. https://doi.org/10.1037/emo0001485](https://www.zotero.org/google-docs/?zRyqN1)

[Fernández, A., Elmer, T., Sádaba, C., García-Manglano, J., & Vanden Abeele, M. (2025). The quality of face-to-face and digitally mediated social interactions: Two experience sampling studies exploring the moderating role of physical location, interaction partner familiarity, and interaction purpose. *Journal of Computer-Mediated Communication*, *30*(2), zmaf004. https://doi.org/10.1093/jcmc/zmaf004](https://www.zotero.org/google-docs/?zRyqN1)

[Fernández, A., Vanden Abeele ,Mariek, Sádaba ,Charo, García-Manglano ,Javier, & and Weinstein, N. (2025). Feeling valued as a conversation-specific relational experience: An examination of Buber’s existential dialogical theory. *The Journal of Positive Psychology*, *0*(0), 1–14. https://doi.org/10.1080/17439760.2025.2481046](https://www.zotero.org/google-docs/?zRyqN1)

[Hall, J. A. (2018). When is social media use social interaction? Defining mediated social interaction. *New Media & Society*, *20*(1), 162–179. https://doi.org/10.1177/1461444816660782](https://www.zotero.org/google-docs/?zRyqN1)

[Langener, A. M., Bringmann, L. F., Kas, M. J., & Stulp, G. (2024). Predicting Mood Based on the Social Context Measured Through the Experience Sampling Method, Digital Phenotyping, and Social Networks. *Administration and Policy in Mental Health and Mental Health Services Research*, *51*(4), 455–475. https://doi.org/10.1007/s10488-023-01328-0](https://www.zotero.org/google-docs/?zRyqN1)

[Rosseel, Y. (2012). lavaan: An R package for structural equation modeling. *Journal of Statistical Software*, *48*(2), 1–26.](https://www.zotero.org/google-docs/?zRyqN1)

[Snijders, T. A. B., & Bosker, R. J. (1999). An Introduction to Basic and Advanced Multilevel Modeling-Sage Publications Ltd (1999).pdf. In *Book*. https://doi.org/10.1136/bcr-2013-009959](https://www.zotero.org/google-docs/?zRyqN1)

[Stadel, M., Stulp, G., Langener, A. M., Elmer, T., Van Duijn, M. A. J., & Bringmann, L. F. (2023). Feedback About a Person’s Social Context—Personal Networks and Daily Social Interactions. *Administration and Policy in Mental Health and Mental Health Services Research*. https://doi.org/10.1007/s10488-023-01293-8](https://www.zotero.org/google-docs/?zRyqN1)
